# Supplementary figures and images for: Spectral dependency of the human pupillary light reflex. Influences of pre-adaptation and chronotype
Source: PLoS One. 2022 Jan 12;17(1):e0253030. doi: 10.1371/journal.pone.0253030 (PMC8754338; doi:10.1371/journal.pone.0253030)

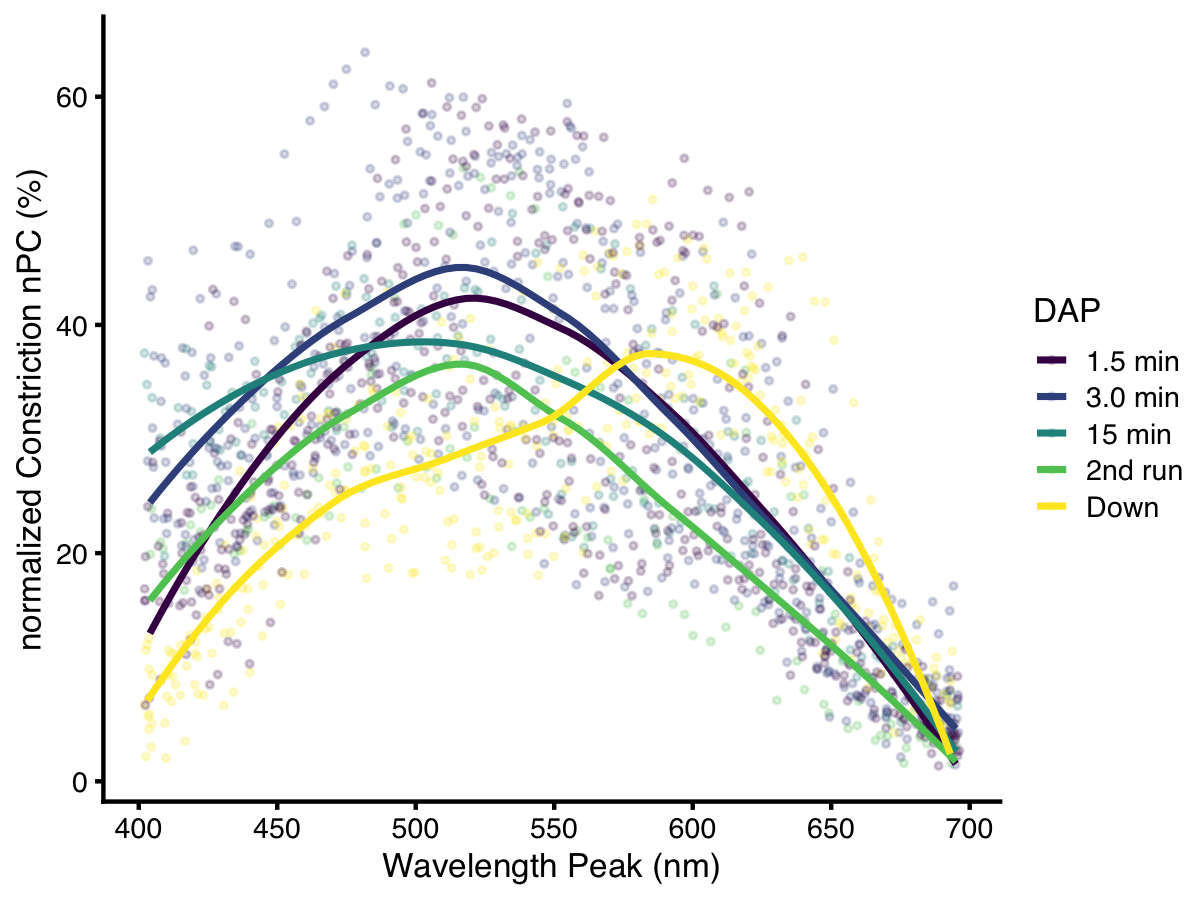

Supplement: S1 Fig — Traces show the mean, LOESS-smoothed nPC vs. wavelength for two subjects, each repeating all shown protocols three times. DAP varied between 1.5, 3, and 15 minutes (lilac, blue, dark green). For comparison, results of a second, consecutive run (also performed three times, light green) of the first protocol is shown, as is the Down protocol (yellow). Points show the nPC values from which the traces were constructed. More information is found in Materials and methods. (TIF) [file pone.0253030.s001.tif]

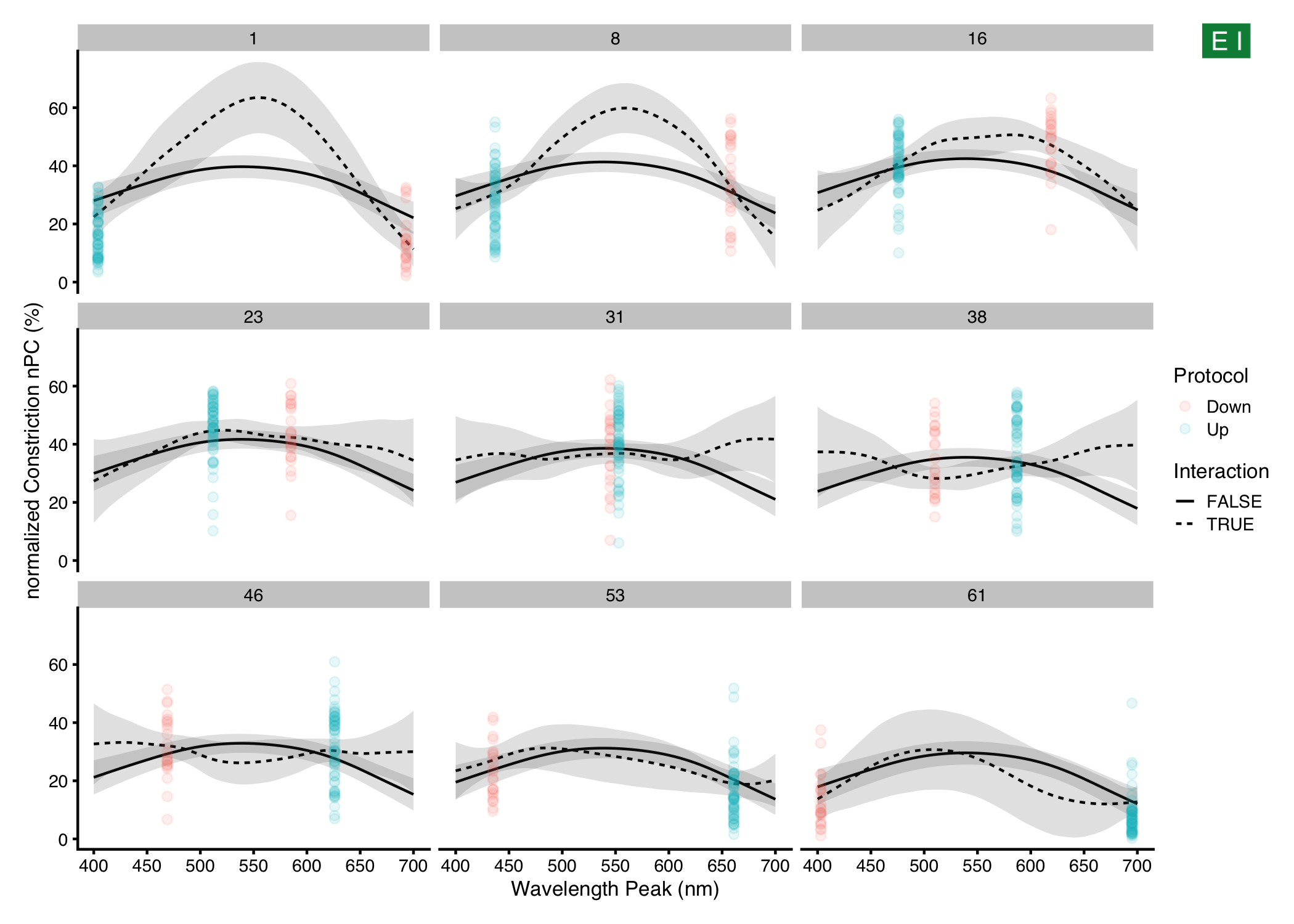

Supplement: S2 Fig — Model prediction for models with (dashed lines), and without an interaction effect (solid lines) of wavelength and series, across the series. The number above each plot indicates the series number. Colored dots represent raw nPC values at the respective series number and wavelength, their color indicates the respective protocol. The positions of points on the x-axis indicate where changes between the two models (lines) should be evaluated. For the model with the interaction effect, it seems as though nPC is increased after reaching the ipRGC peak sensitivity (at about 490 nm for a young adult), but not strongly and not for long. (TIF) [file pone.0253030.s002.tif]

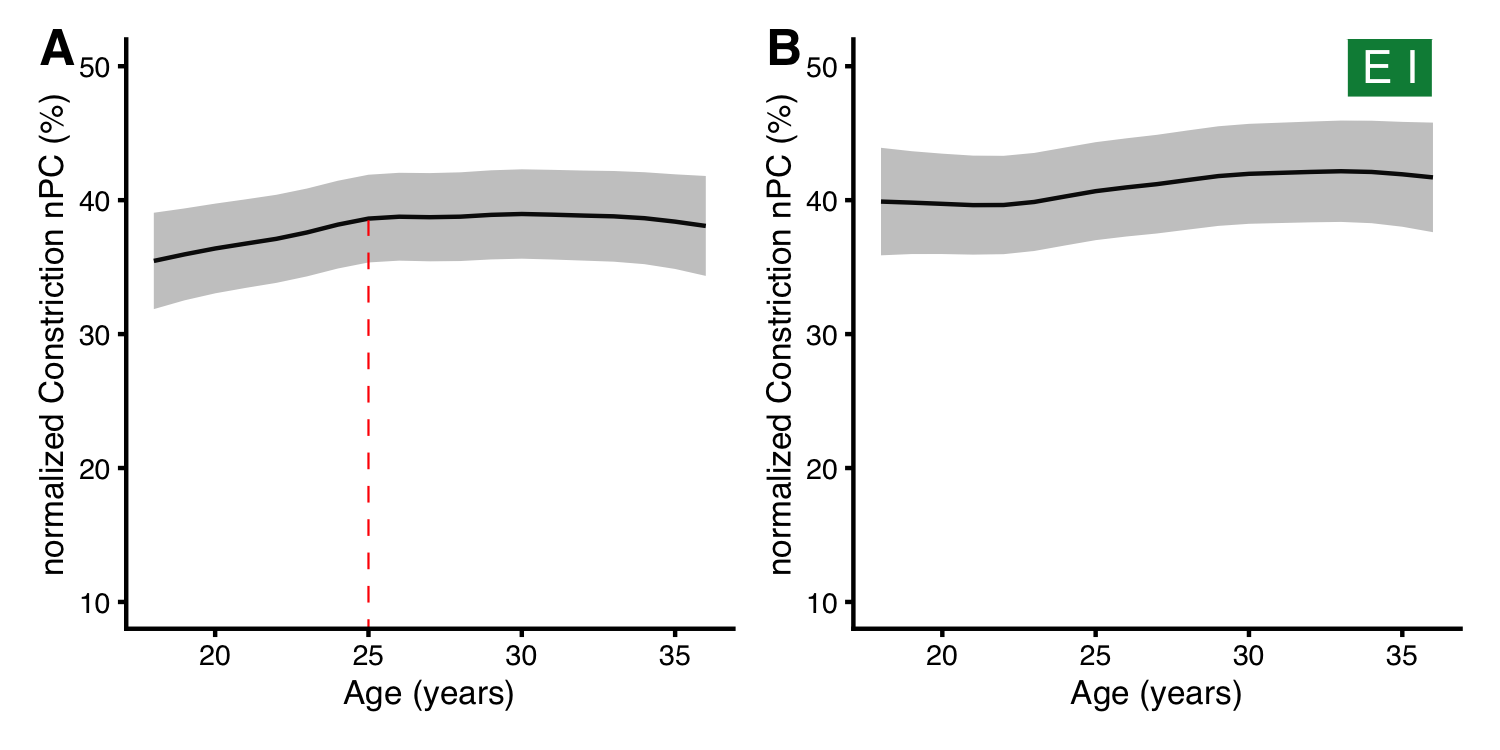

Supplement: S3 Fig — (A) Model predictions how nPC depends on age, when all other predictors (basic model) are held constant at an average level. (B) Like (A), but for the model with all other dependencies in Experiment I included. Here, age seems to affect nPC with a more continuous rise over the age range. (TIF) [file pone.0253030.s003.tif]

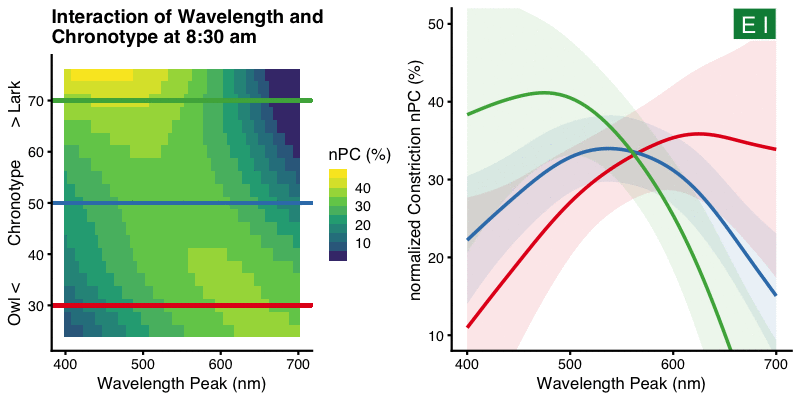

Supplement: S4 Fig — Model predictions show time-of-day values at half-hour points from 8:30 am to 7:30 pm. Left: False-color model predictions for hourly nPC values depending on wavelength (x-axis) and chronotype (y-axis), when all other predictors (basic model) are held constant at an average level. Horizontal lines show where the traces from the right image are taken from. Right: Model predictions for hourly nPC values depending on wavelength for three chronotypes: Larks (green traces, CT score 70), Owls (red traces, CT score 30), and Neutral types (blue traces, CT score 50). Ribbons show the 95% confidence interval for the predicted mean values. (GIF) [file pone.0253030.s004.gif]

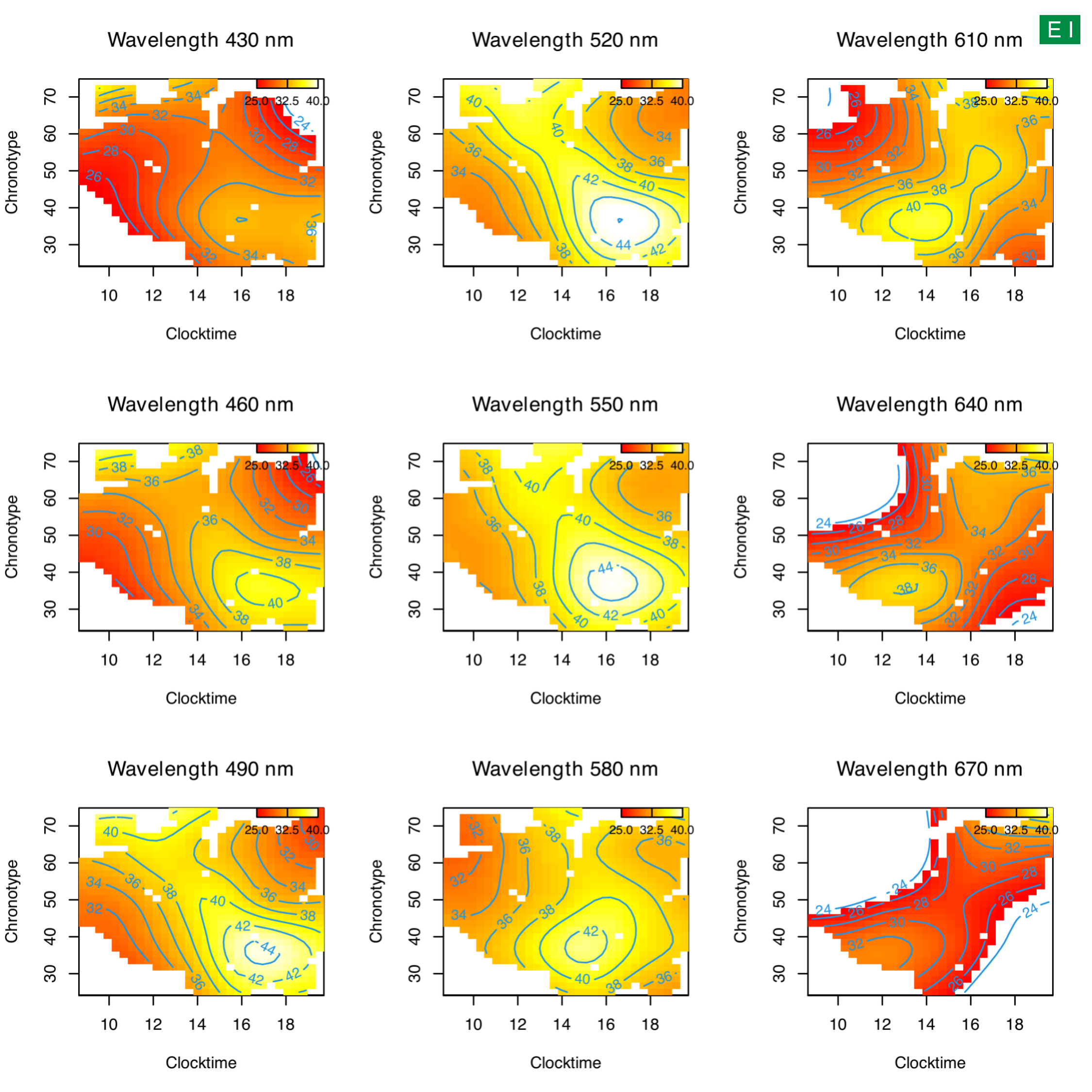

Supplement: S5 Fig — False-color contour-line graphs of nPC model predictions for several wavelengths between 430 and 670 nm (color scale shown in the inset at the upper right corner of each plot). All plots are scaled equally. Each plot visualizes nPC depending on time of day (clock time in 24h values, x-axis) and chronotype (higher values are morning types, lower values evening types). Plots were created with the vis.gam() function in R, with the too.far argument set to 0.1. The too.far argument excludes grid points from the plot, when points are not represented by variable combinations close enough to actual data. Thereby, too.far is a measure of accepted extrapolation, scaled from 0 to 1. (TIF) [file pone.0253030.s005.tif]

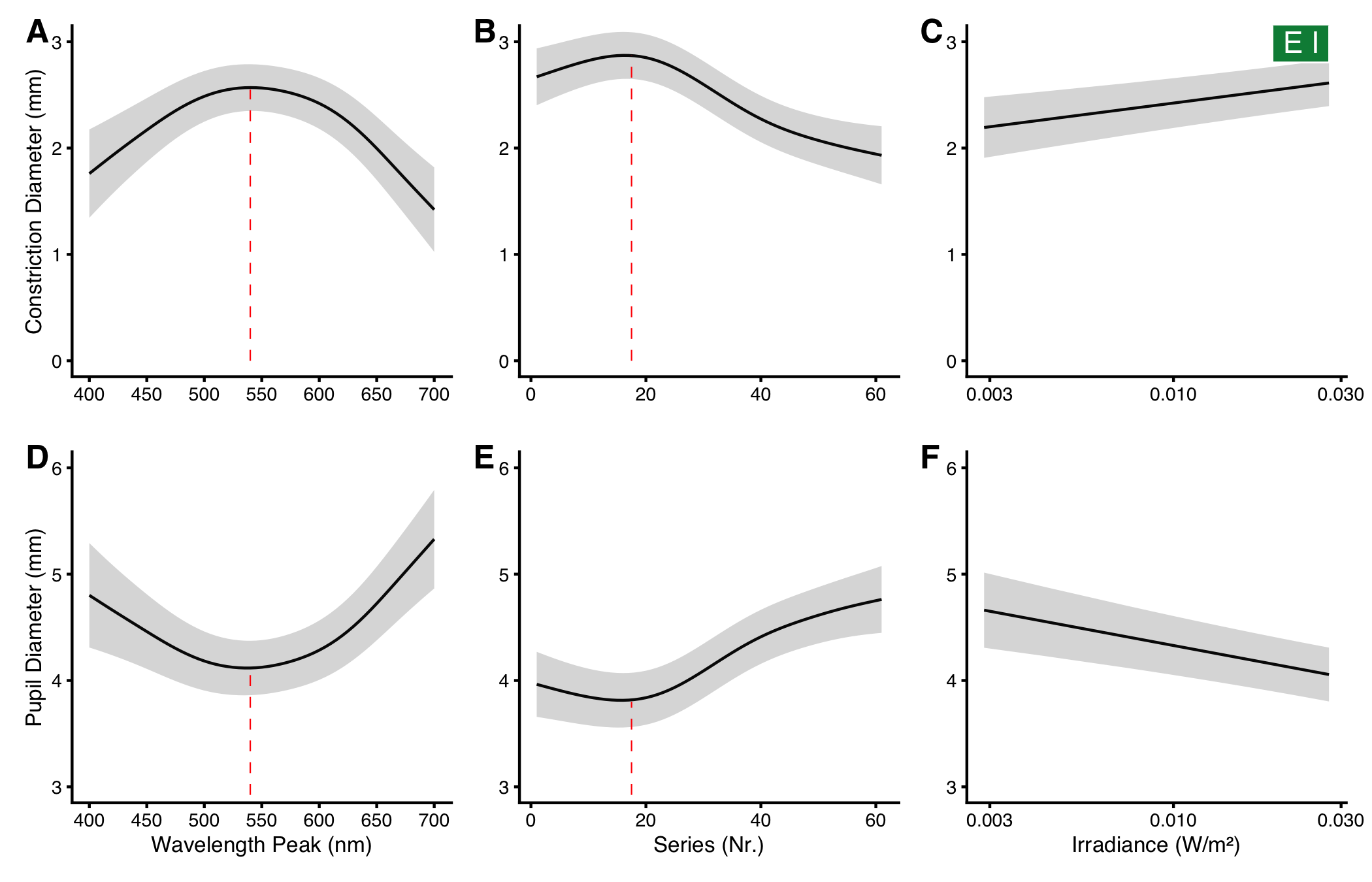

Supplement: S6 Fig — Model predictions for response variables other than nPC, versus three main predictors (wavelength, series, and irradiance), when all other predictors are held constant at their average level. Traces show the model prediction for the mean, ribbons its 95% confidence interval. Red dotted lines show particular predictor values for the plotted relationship. (A / D) Response vs. stimulus peak wavelength. (B / E) Response vs. series number of light steps. (C) Response vs. stimulus irradiance. The x-axis scaling reflects the logarithmic transformation of irradiance. (TIF) [file pone.0253030.s006.tif]

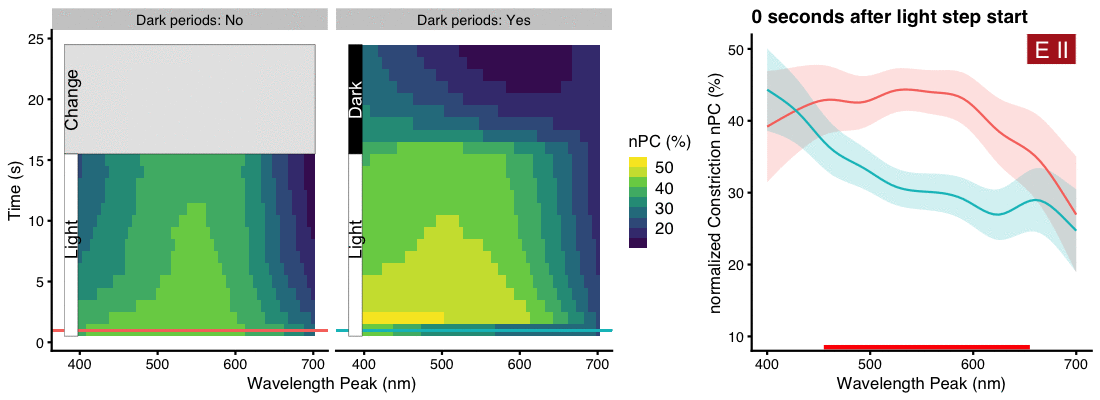

Supplement: S7 Fig — Left: False-color model predictions for nPC depending on wavelength (x-axis) and time (y-axis), for settings with, or without, periods of darkness between wavelength steps. All other predictors are held constant at an average level. Horizontal lines show where the traces from the right image are taken from. Right: Model predictions for the nPC vs. wavelength over time. Ribbons show the 95% confidence interval for the predicted means. Blue lines represent the setting when periods of darkness are present between light steps, red lines when not. The red horizontal line above the x-axis shows where the difference between the two settings is significant (5% level). (GIF) [file pone.0253030.s007.gif]

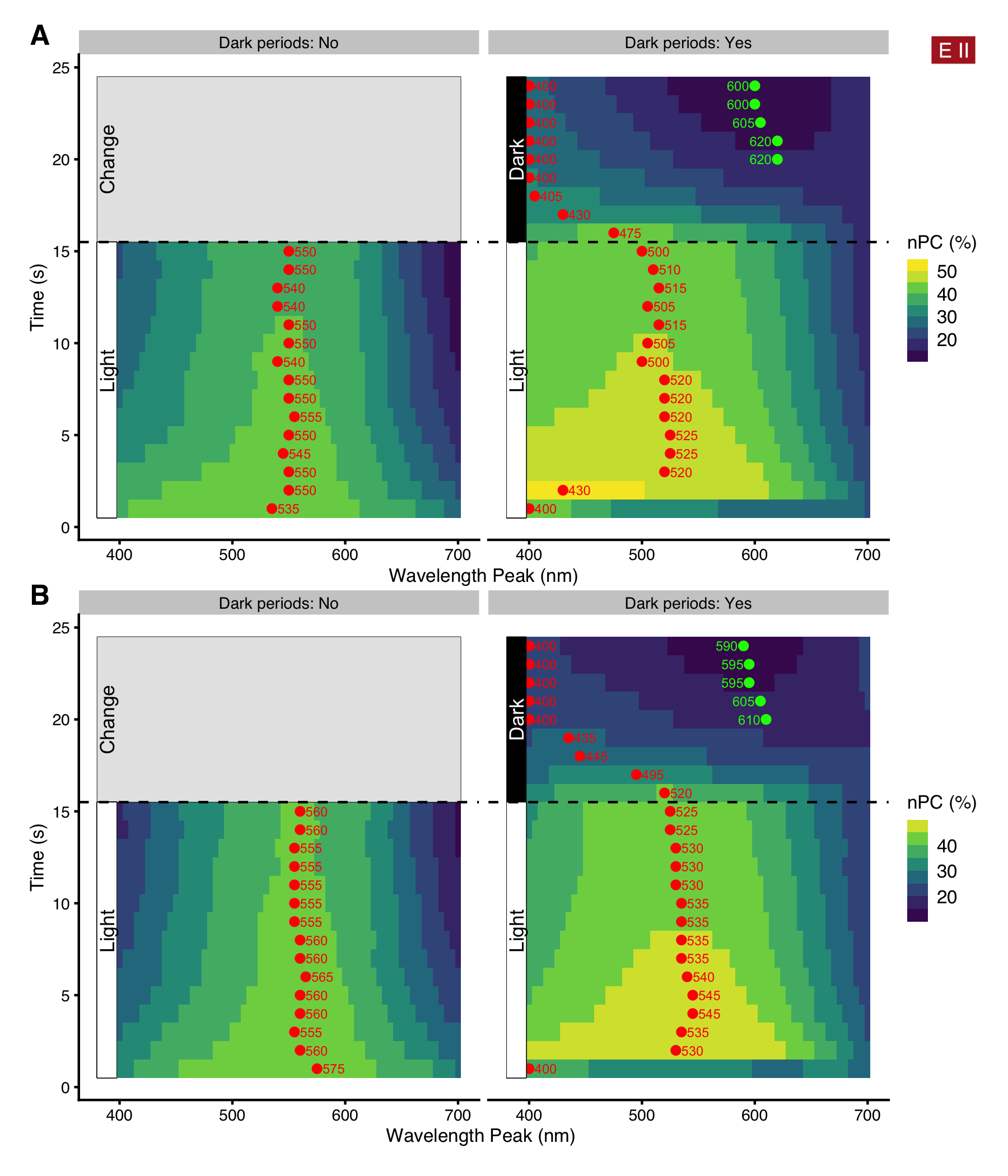

Supplement: S8 Fig — (A) False-color graph of model predictions for the nPC’s dependence on wavelength (x-axis) and time (y-axis) for settings with, or without, periods of darkness between wavelength steps. All other predictors (basic model) are held constant at their average. Red dots show the peak nPC value for each second, the value right next to it the respective peak wavelength. Green dots and values show the respective trough for nPC. The trough is at 700 nm where green dots are not shown. (B) Like (A), but for the model without irradiance as predictor. (TIF) [file pone.0253030.s008.tif]

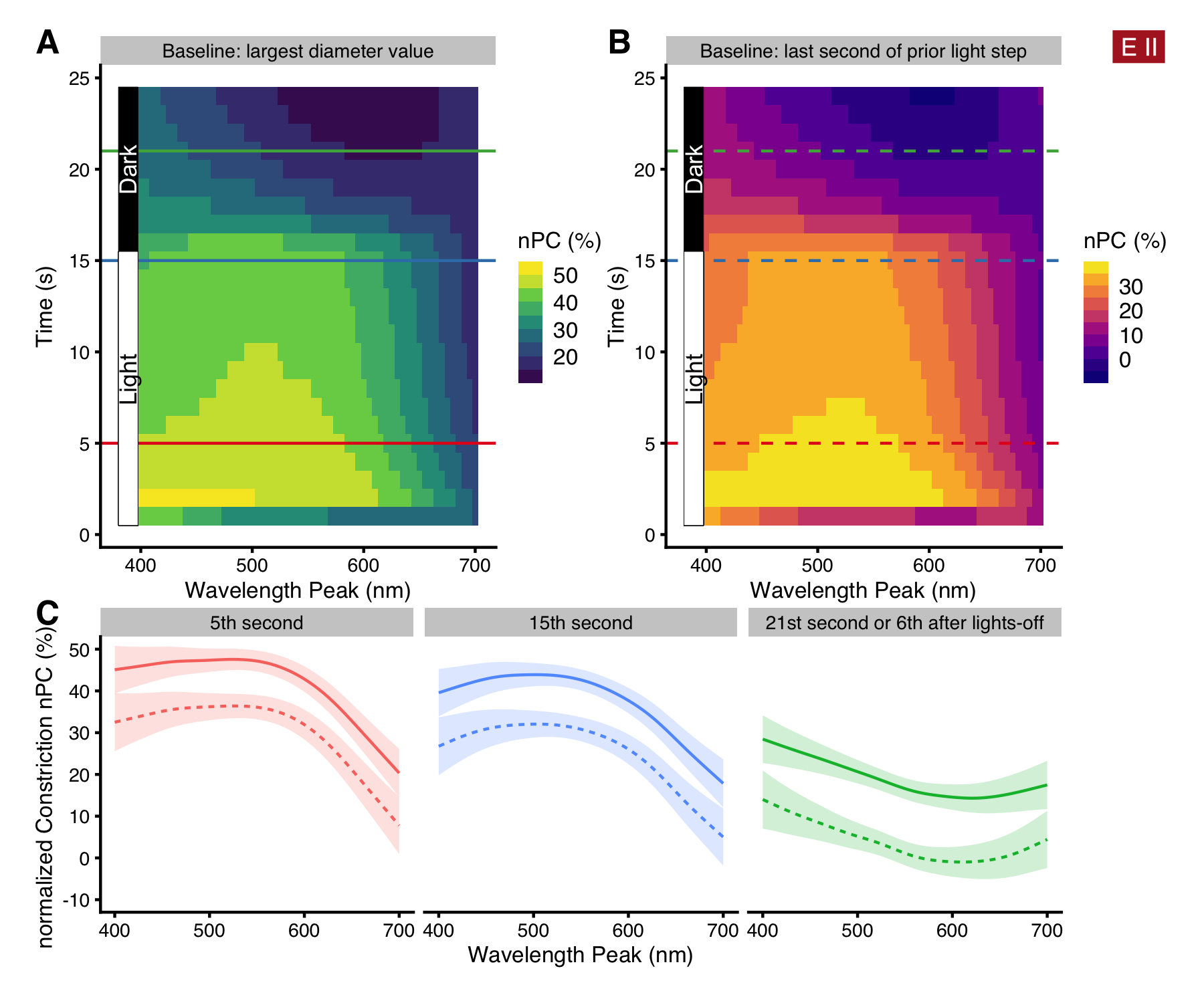

Supplement: S9 Fig — (A) False-color graph of model predictions for the nPC’s dependence on wavelength (x-axis) and time (y-axis). All other predictors (basic model) are held constant at their average. Horizontal lines show where the respective traces shown in part (C) are taken from. (B) Same as in (A) except that the baseline for nPC calculation (Eq 1) is taken from the last second of the respective previous light step. (B) The graph serves to check whether and how the single baseline value per protocol (shown in A) changes the model prediction. (C) Model predictions for the nPC vs. wavelength at three points in time after light step onset: in the 5th second (red), 15th second (blue), and 21st second (green); the latter case is also the sixth seconds after lights-off. Ribbons show the 95% confidence interval for the predicted means. Full lines represent nPC values when baseline pupil values are taken from each protocol’s largest pupil diameter, dotted lines when baseline pupil values are taken from the last second of the respective prior wavelength-step. (TIF) [file pone.0253030.s009.tif]

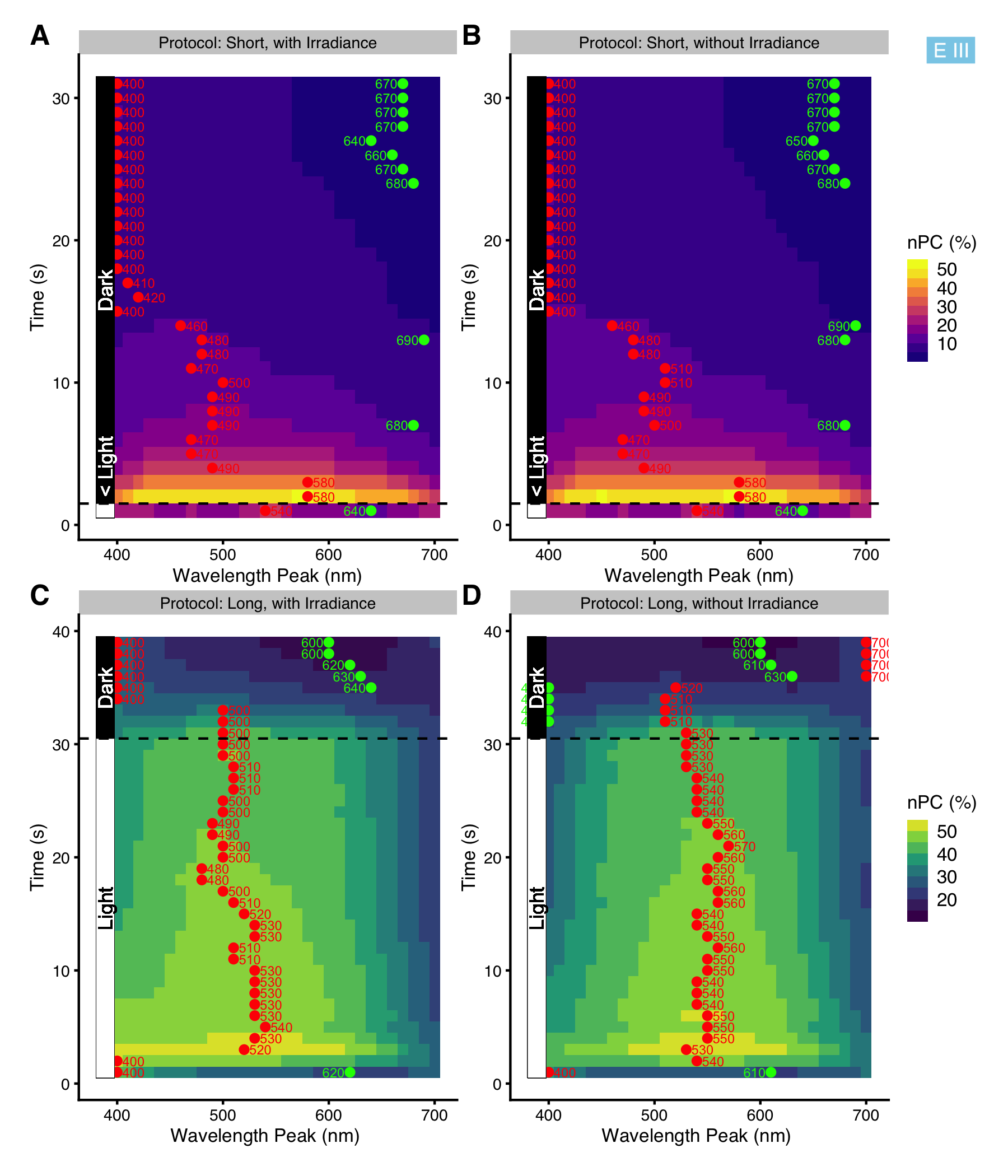

Supplement: S10 Fig — (A) False-color graph of model predictions for the nPC’s dependence on wavelength (x-axis) and time (y-axis) for the protocols with one second of light, followed by thirty seconds of darkness, and with irradiance as the predictor. All other predictors (basic model) are held constant at their average. Red dots show the peak nPC value for each second, the value right next to it the respective peak wavelength. Green dots and values show the respective trough for nPC. The trough is at 700 nm where green dots are not shown (e.g. between 2 and 30 seconds). The baseline for nPC calculation (Eq 1) is taken from the last second of the respective previous light step, since this baseline led to a preferable model in terms of model diagnostics. (B) Like (A), but for the model without irradiance as the predictor. (C) Like (A), but for protocols with thirty seconds of light, followed by nine seconds of darkness with irradiance as a predictor. The baseline for nPC is as described in Materials and methods. (D) Like (C), but for the model without irradiance as the predictor. (TIF) [file pone.0253030.s010.tif]

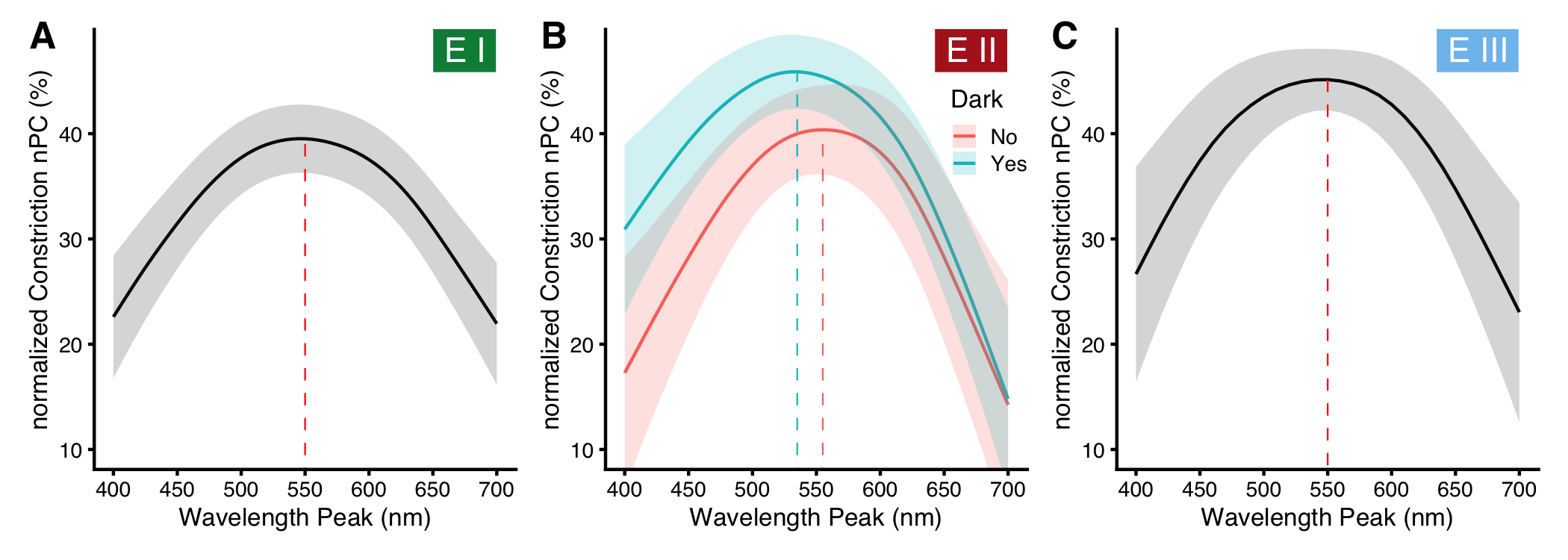

Supplement: S11 Fig — Normalized pupillary constriction (nPC) as depending on wavelength, when all other predictors are held at an average, constant level. Traces show the model prediction for the mean, ribbons its 95% confidence interval. Dotted lines show the respective peak. (A) through (C) show dependencies in Experiment I, II, and III, respectively. They can be compared to the model results which include irradiance in Fig 5A1, 5B1 and 5C1. (TIF) [file pone.0253030.s011.tif]

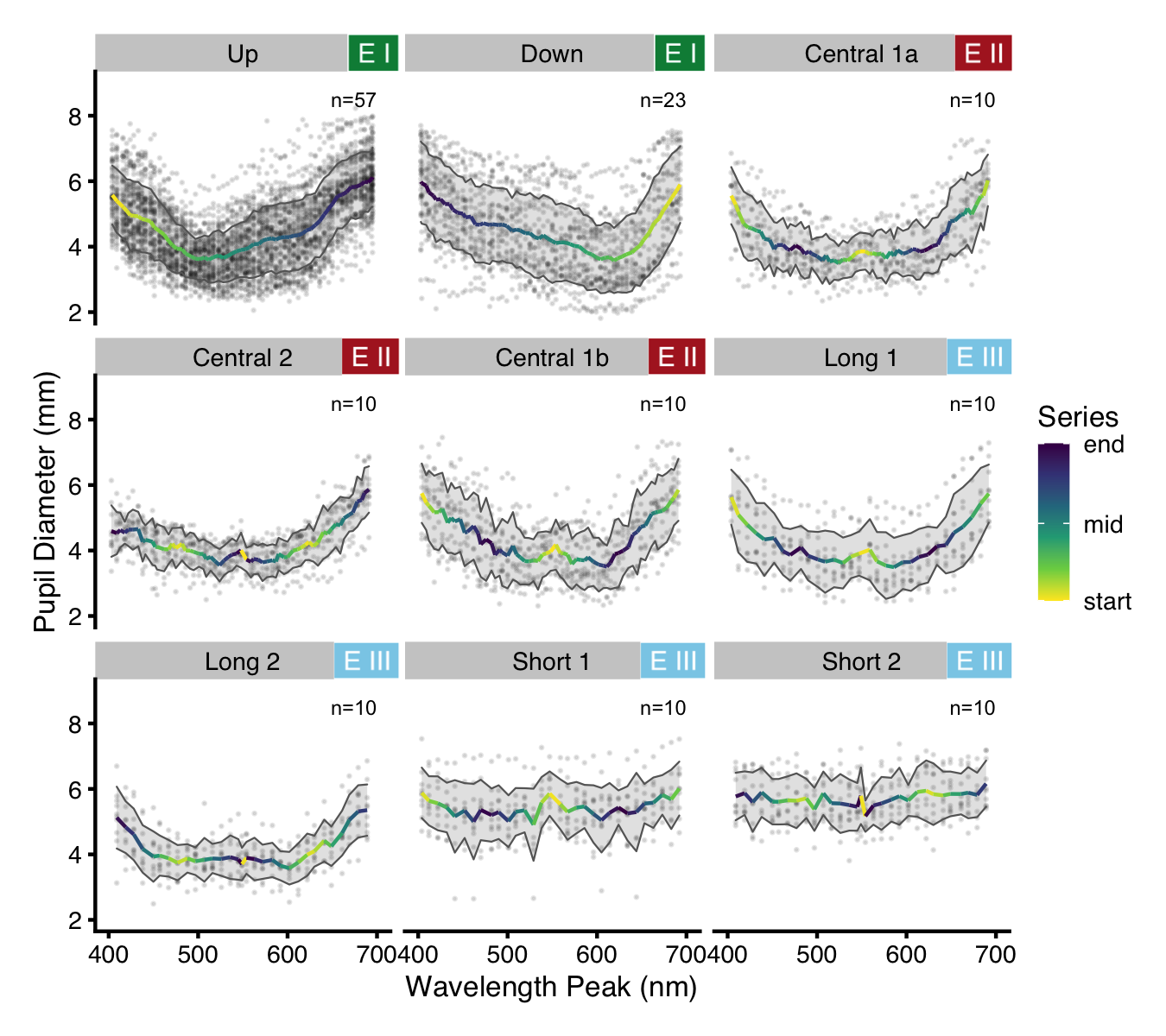

Supplement: S12 Fig — (A) Pupil diameter plotted against wavelength, for each of the nine protocols. The color scale shows at which point in the series a specific wavelength was presented; light yellow represents early in the series. For all but the Short protocols, points represent the average nPC during the respective last five seconds of a light step. In the two Short protocols, points represent the average nPC during the sixth second after lights-off (or sevenths second after lights-on). Traces show the mean nPC, ribbons its standard deviation. The number in the upper right corner of each plot shows the corresponding sample size. (TIF) [file pone.0253030.s012.tif]

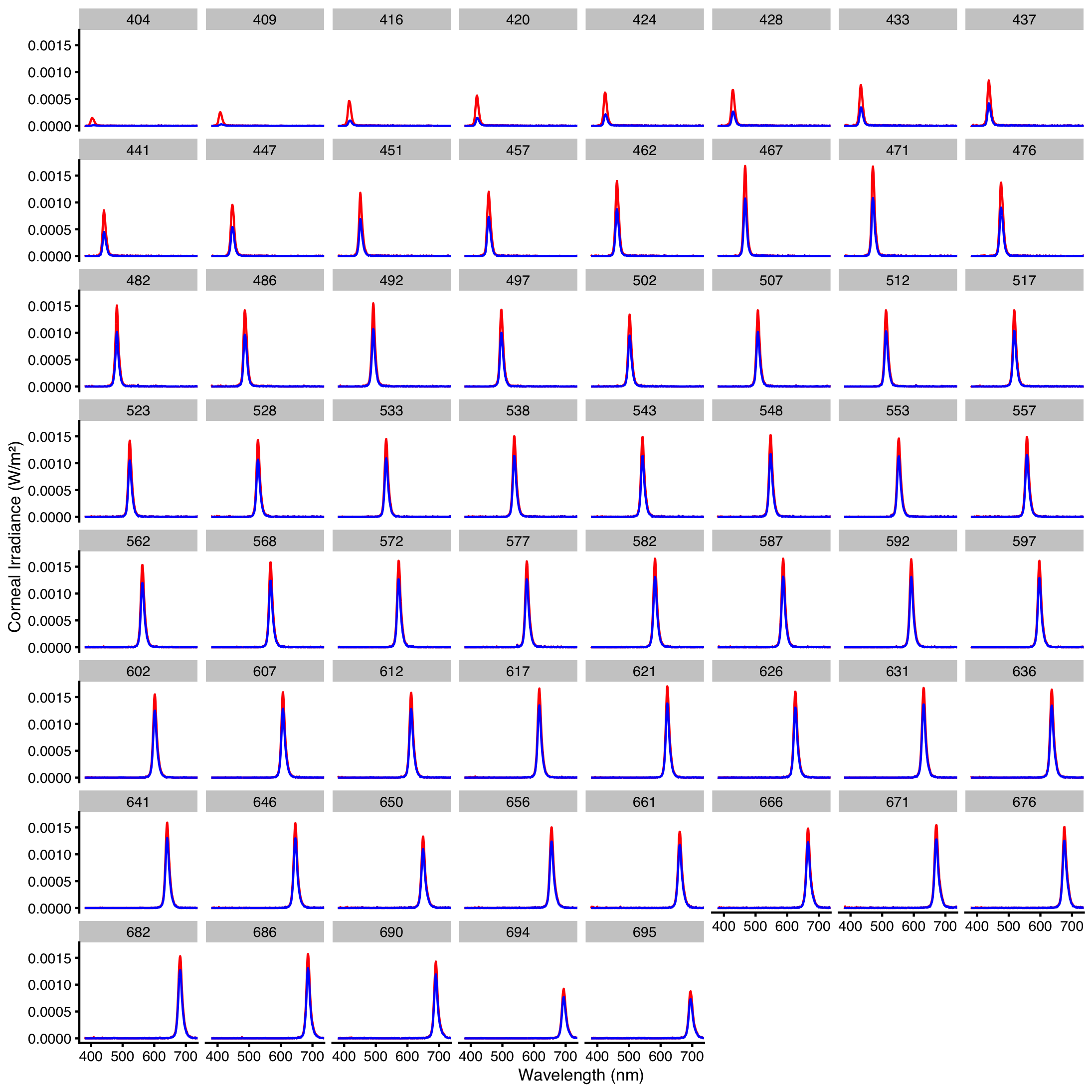

Supplement: S13 Fig — Corneal irradiance (W/m2) vs. wavelength, labelled by wavelengths peaks (404–695 nm) above each subgraph. The x-axis on the bottommost plots and the y-axis on the left apply to all plots in their respective row and column. Blue and red traces show the spectral measurements with and without an estimate of prereceptoral filtering, respectively. Wavelength peaks above each plot state the peak for the raw stimulus measurement. All displayed values are based on spectral irradiance measurements with a field-of-view restriction according to the CIE S 026 standard [32]. In our case, these measurements are 24% lower than those of the unobstructed sensor diffusor. (TIF) [file pone.0253030.s013.tif]

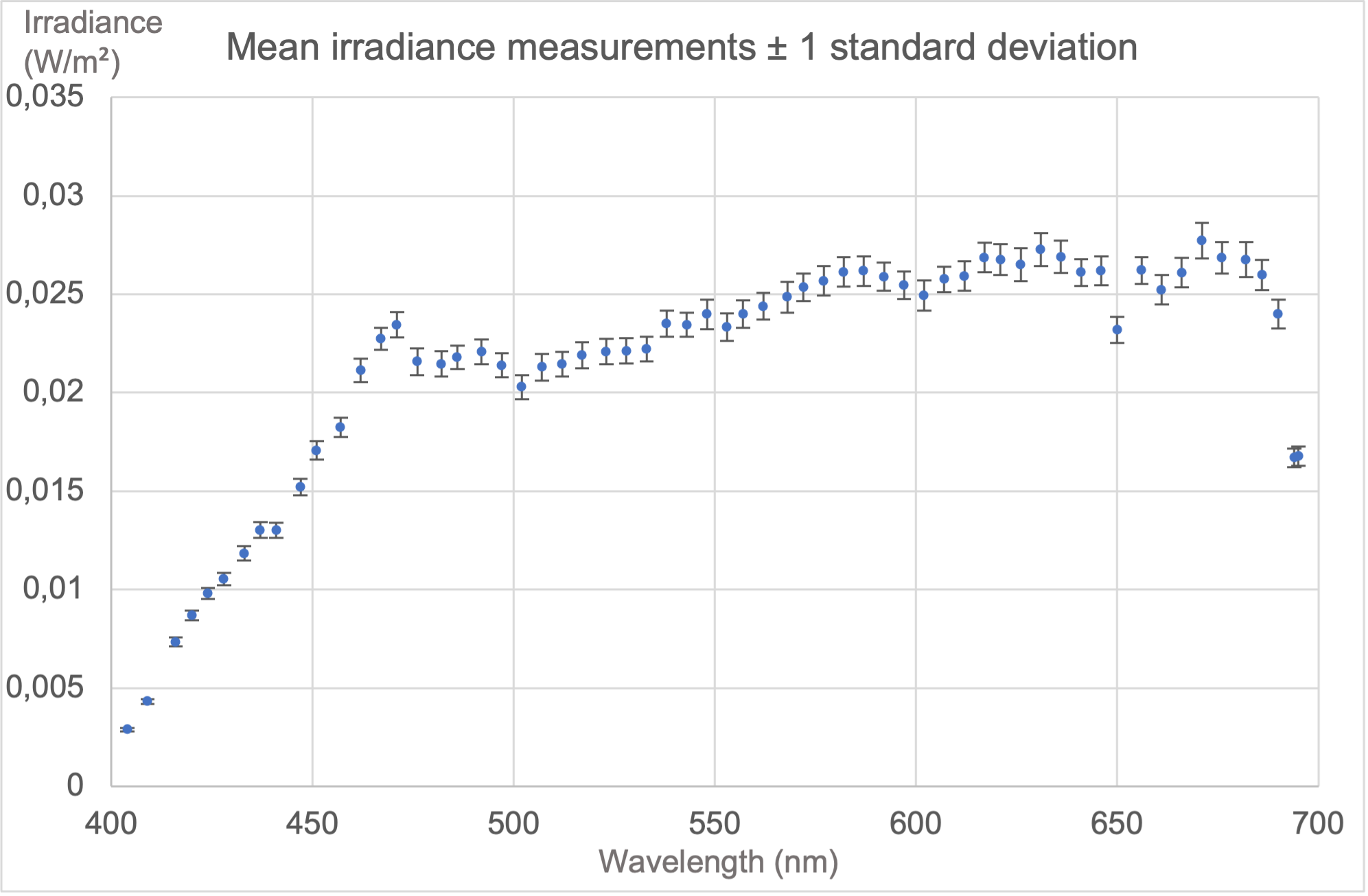

Supplement: S14 Fig — Corneal irradiance (W/m2) against wavelength for all wavelength peaks, with their respective standard deviation across multiple measurements. All displayed values are based on spectral irradiance measurements with a field-of-view restriction according to the CIE S 026 standard [32]. In our case, these measurements are 24% lower than those of the unobstructed sensor diffusor. (TIF) [file pone.0253030.s014.tif]

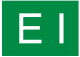

Supplement: S3 File — Zip file containing several files necessary to replicate the statistical analysis and generation of graphics. Besides some external tables, an R-function file, and three pictures to mark the respective experiment in graphs, the zip file consists of nine R-Markdown scripts. One of these is used to set up the data prior to analysis. The necessary data can be downloaded from the Open Science Framework [39]. Five of the R-Markdown files are for analysis of Experiment I, II, III Short, III Long, and the pooled data. The eighth file is for graphics generation. The second-to-last file takes a sample of ten random participants from the first experiment for every protocol and builds the base model from this subset. This sample analysis is to show that the main dependencies of wavelength and series can come from a smaller sample. An html file with the same file name shows an example. The last file is for calculating estimates for prereceptoral filtering and using those estimates to calculate irradiance and photon density values from the spectral irradiance measurements. (ZIP) [file pone.0253030.s017.zip › E1.png]

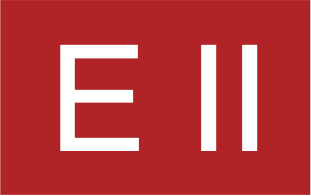

Supplement: S3 File — Zip file containing several files necessary to replicate the statistical analysis and generation of graphics. Besides some external tables, an R-function file, and three pictures to mark the respective experiment in graphs, the zip file consists of nine R-Markdown scripts. One of these is used to set up the data prior to analysis. The necessary data can be downloaded from the Open Science Framework [39]. Five of the R-Markdown files are for analysis of Experiment I, II, III Short, III Long, and the pooled data. The eighth file is for graphics generation. The second-to-last file takes a sample of ten random participants from the first experiment for every protocol and builds the base model from this subset. This sample analysis is to show that the main dependencies of wavelength and series can come from a smaller sample. An html file with the same file name shows an example. The last file is for calculating estimates for prereceptoral filtering and using those estimates to calculate irradiance and photon density values from the spectral irradiance measurements. (ZIP) [file pone.0253030.s017.zip › E2.png]

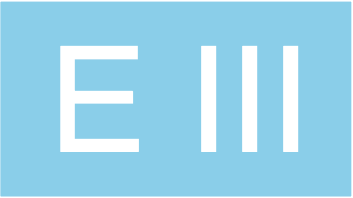

Supplement: S3 File — Zip file containing several files necessary to replicate the statistical analysis and generation of graphics. Besides some external tables, an R-function file, and three pictures to mark the respective experiment in graphs, the zip file consists of nine R-Markdown scripts. One of these is used to set up the data prior to analysis. The necessary data can be downloaded from the Open Science Framework [39]. Five of the R-Markdown files are for analysis of Experiment I, II, III Short, III Long, and the pooled data. The eighth file is for graphics generation. The second-to-last file takes a sample of ten random participants from the first experiment for every protocol and builds the base model from this subset. This sample analysis is to show that the main dependencies of wavelength and series can come from a smaller sample. An html file with the same file name shows an example. The last file is for calculating estimates for prereceptoral filtering and using those estimates to calculate irradiance and photon density values from the spectral irradiance measurements. (ZIP) [file pone.0253030.s017.zip › E3.png]
