# Supplementary material for: Spectral dependency of the human pupillary light reflex. Influences of pre-adaptation and chronotype
Source: PLoS One. 2022 Jan 12;17(1):e0253030. doi: 10.1371/journal.pone.0253030 (PMC8754338; doi:10.1371/journal.pone.0253030)
Supplement: S1 File — Zip file containing nine html files with results from R-Markdown scripts. The scripts are one each for the nine protocols used across the experiments. Since the raw measurement data are not part of the provided supplements, we included one exemplary result file for each protocol instead of the script itself. These html files show the code, as well as the output for the example participant. (ZIP) [file pone.0253030.s015.zip › E1_Up.html]

R Pupil


Code 

- Show All Code
- Hide All Code
- Download Rmd

# R Pupil

Mit diesem Dokument werden aus den Dikablis Pupillenrohdaten mit dem Hochschule-München Lichtlabor(D113)-Setup auswertungsfähige Pupillendaten extrahiert. Die Auswertung ist gültig für das “Programm 4” aus dem Lightdome-Steuerrechner und für die “neue” Umrechnungsmethode von Pixeln zu mm, bei der vor Aufzeichnung ein Referenzmaß auf Augenebene im Kamerabild gezeigt wird. Dieses Programm beginnt mit einer Dunkeladapationsperiode von 90 Sekunden. Danach werden in ca. 15 Sekunden-Schritten die Wellenlängen von 400-700 nm in 5 nm Schritten abgefahren.

Um den Code für eine neue Person anzupassen, sind die folgenden Eingriffe notwendig. Zeilenangaben beziehen sich auf das \*.Rmd-File:

1. Zeile 37 -> Einfügen des korrekten Dateinamens / Pfad
2. Zeile 45 -> Versuchspersonencode einfügen
3. Zeilen 116-126 -> Prüfung, ob die Kriterien zur Messwertaussortierung bei Zirkularität, absolutem Abschnitt, und dem Schlussschnitt in Ordnung sind
4. Zeilen 209-212 -> Nach Prüfung, ob die Tal-Erkennung funktioniert, Angabe des korrekten Index für den Referenzzeitpunkt. Sollte das beim linken Auge nicht möglich sein, muss der unterste Code-Chunk aus der Datei manuell hoch kopiert werden. Sollte der erste Beleuchtungszeitpunkt nicht erkannt werden, kann ein späterer Zeitpunkt angegeben werden - in diesem Fall muss spezifiziert werden, um wie viele Schritte der Programmstart zurückliegt.
5. Zeile 426/427 -> Die Auswahl, welches Auge schlussendlich für den Datenexport verwendet wird, wird standardmäßig automatisch vollzogen anhand der Pupille mit dem meisten Datenpunkten nach der Bereinigung. Sollte diese Auswahl nicht in Ordnung sein, muss hier manuell “R” oder “L” eingetragen werden.

Im folgenden Abschnitt werden alle notwendigen Bibliotheken geladen, sowie die Rohdaten eingelesen. Der Abschnitt endet mit einer Darstellung der Rohdaten der Pupillen, sowie der Zirkulariät.


```
library(tidyverse)
library(anytime)
library(signal)
library(ggplot2)
library(cowplot)
library(ggpmisc)
library(knitr)
library(readxl)

setwd(dirname(rstudioapi::getActiveDocumentContext()$path)) # Setzen des Arbeitsverzeichnisses auf gleiches Verzeichnis wie Script 

pup <- read.table("../data_input/Up/KG110502_4. Messung Pr4 17.08.2020 131803_CsvData.txt", sep = "\t", header = TRUE, stringsAsFactors=F) # Einlesen der Daten Pupille
prog <- read.table("../data_input/program_data_ld.csv", dec = ",", sep = ";", header = TRUE, stringsAsFactors=F) # Einlesen der Programmdaten
corf <- read_excel("../Übersicht_VP.xlsx", sheet = "Uebersicht_VP", na="NA") #load in the file with all individual codes # Einlesen der Korrekturfaktoren

#Auswählen der notwendigen Spalten und Spalten umbenennen
#Umrechnen des UTC-Zeitstempels in lokale Zeit
#Berechnung des Pupillendurchmessers (nur für Referenzmaß - Die Korrekturfaktoren aller Probanden werden in einer separaten Datei gespeichert)

Code <- "KG110502_2"

dir.create(paste("../figures/", Code, sep=""))

pup <- select(pup, rec_time, UTC, Dikablis.Professional_Eye.Data_Original_Left.Eye_Pupil.Area, Dikablis.Professional_Eye.Data_Original_Left.Eye_Pupil.Height, Dikablis.Professional_Eye.Data_Original_Left.Eye_Pupil.Width, Dikablis.Professional_Eye.Data_Original_Right.Eye_Pupil.Area, Dikablis.Professional_Eye.Data_Original_Right.Eye_Pupil.Height, Dikablis.Professional_Eye.Data_Original_Right.Eye_Pupil.Width) 

pup <- tibble(pup)

colnames(pup) <- c("time", "UTC", "La", "Lh", "Lw", "Ra", "Rh", "Rw")

pup$UTC <- anytime(pup$UTC/1000)

pup <- separate(pup, time, c("h", "m", "s"), sep = ":", convert = TRUE)
pup$time <- pup$s + pup$m*60 + pup$h*60*60

corf_L <- corf$Korr_Links[corf$Code == Code]

corf_R <- corf$Korr_Rechts[corf$Code == Code]

pup$Ld <- 2*sqrt(pup$La/pi)/corf_L

pup$Rd <- 2*sqrt(pup$Ra/pi)/corf_R

#Aufteilen in Pupille rechtes und linkes Auge

pupR <- select(pup, "time", "UTC", "Rd", "Ra", "Rh", "Rw")
pupR <- dplyr::filter(pupR, !is.na(Ra))
colnames(pupR) <- c("time", "UTC", "dia", "area", "height", "width")

pupL <- select(pup, "time", "UTC", "Ld", "La", "Lh", "Lw")
pupL <- dplyr::filter(pupL, !is.na(La))
colnames(pupL) <- c("time", "UTC", "dia", "area", "height", "width")

#Darstellung der Ergebnisse im Plot
# Rohdaten
Roh_plot <- ggplot() + 
  geom_line(data = pupL, aes(x = time, y = dia, col = "Left")) +
  geom_line(data = pupR, aes(x = time, y = dia, col = "Right"), alpha = 0.6) +
  geom_text(aes(label = paste("n=1, ", Code, ", Up", sep=""), x = 50, y= 8), size = 2.5)+
  theme_cowplot(font_size = 8, font_family = "sans")+
  scale_color_brewer(palette = "Set1", "Eye")+
  ggtitle("Pupil-diameter data unchanged")+
  ylab("Pupil diameter / mm") +
  xlab("Time / seconds")+
  theme(legend.position = c(0.9,.1),legend.background = element_rect(fill="white", size = 0.5))

Roh_plot
ggsave("Pupil_Raw.pdf", path = paste("../figures/", Code, sep=""), width=10, height = 5)
```


```
# Zirkularität
Roh_plot2 <- ggplot() + 
  geom_line(data = pupL, aes(x = time, y = width/height, col = "Left")) +
  geom_line(data = pupR, aes(x = time, y = width/height, col = "Right"), alpha = 0.6) +
  geom_text(aes(label = paste("n=1, ", Code, ", Up", sep=""), x = 50, y= 1.01), size = 2.5)+
  theme_cowplot(font_size = 8, font_family = "sans")+
  scale_color_brewer(palette = "Set1", "Eye")+
  ggtitle("Pupil-circularity")+
  ylab("Pupil diameter / mm") +
  xlab("Time / seconds")+
  theme(legend.position = c(0.9,.1),legend.background = element_rect(fill="white", size = 0.5))

Roh_plot2
ggsave("Pupil_Raw2.pdf", path = paste("../figures/", Code, sep=""), width=10, height = 5)
```


```
NA
NA
```


Im kommenden Abschnitt werden Artefakte und unplausible Messwerte entfernt. Zudem wird die Kurve geglättet.


```
#Entfernung von Ausreissern und Werten bei denen die Pupille eine zirkularität < 0,7 aufweist, zudem wird die letzten 2 Sekunden der Messung entfernt

pupRf <- pupR[1:(length(pupR$time)-120),]
pupRf <- dplyr::filter(pupRf, dia > 2) # Entfernung von Werten kleiner X mm
pupRf <- dplyr::filter(pupRf, dia < 9) # Entfernung von Werten größer 8 mm
pupRf <- dplyr::filter(pupRf, width/height > 0.70 & width/height > 0.70) # Entfernung von Werten für die die Zirkularität kleiner als 0,7 ausfällt
pupRf$dia_sm <- sgolayfilt(pupRf$dia, p=3, n=31) # Glättung der Kurve mittels Savitzky-Golay Filter

pupLf <- pupL[1:(length(pupL$time)-120),]
pupLf <- dplyr::filter(pupLf, dia > 2) # Entfernung von Werten kleiner X mm
pupLf <- dplyr::filter(pupLf, dia < 9) # Entfernung von Werten größer 8 mm
pupLf <- dplyr::filter(pupLf, width/height > 0.70 & width/height > 0.70) # Entfernung von Werten für die die Zirkularität kleiner als 0,7 ausfällt
pupLf$dia_sm <- sgolayfilt(pupLf$dia, p=3, n=31) # Glättung der Kurve mittels Savitzky-Golay Filter

Smooth_plot_test <- ggplot() + 
  geom_line(data = pupLf, aes(x = time, y = dia, col = "unsmoothed")) +
  geom_line(data = pupLf, aes(x = time, y = dia_sm, col = "sg n=31"), alpha = 0.6) +
    geom_line(data = pupLf, aes(x = time, y = sgolayfilt(dia, p=3, n=61), col = "sg n=61"), alpha = 0.6) +
    geom_line(data = pupLf, aes(x = time, y = sgolayfilt(dia, p=3, n=91), col = "sg n=91"), alpha = 0.6) +
  geom_text(aes(label = paste("n=1, ", Code, ", Up", sep=""), x = 50, y= 8), size = 2.5)+
  theme_cowplot(font_size = 8, font_family = "sans")+
  scale_color_brewer(palette = "Set1", "Eye")+
  ggtitle("Pupil-diameter data smoothed, test settings")+
  ylab("Pupil diameter / mm") +
  xlab("Time / seconds")+
  theme(legend.position = c(0.9,.1),legend.background = element_rect(fill="white", size = 0.5))+
  coord_cartesian(xlim = c(250, 275), ylim = c(2,8))

Smooth_plot_test
ggsave("Pupil_Smooth_test.pdf", path = paste("../figures/", Code, sep=""), width=10, height = 5)
```


```
Smooth_plot <- ggplot() + 
  geom_line(data = pupLf, aes(x = time, y = dia_sm, col = "Left")) +
  geom_line(data = pupRf, aes(x = time, y = dia_sm, col = "Right"), alpha = 0.6) +
  geom_text(aes(label = paste("n=1, ", Code, ", Up", sep=""), x = 50, y= 8), size = 2.5)+
  theme_cowplot(font_size = 8, font_family = "sans")+
  scale_color_brewer(palette = "Set1", "Eye")+
  ggtitle("Pupil-diameter data smoothed")+
  ylab("Pupil diameter / mm") +
  xlab("Time / seconds")+ 
  coord_cartesian(ylim = c(2,8))+
  theme(legend.position = c(0.9,.1),legend.background = element_rect(fill="white", size = 0.5))

Smooth_plot
ggsave("Pupil_Smooth.pdf", path =  paste("../figures/", Code, sep=""), width=10, height = 5)
```


```
NA
NA
```


Im nächsten Abschnitt erfolgt die Zuordnung einzelner Zeitabschnitte zu den Wellenlängen.


```
#Bildung der 1. und 2. Ableitung. Lineare Transformation, damit die Überlagerung mit dem Pupillendurchmesser lesbar bleibt.

pupLf$dia_sm1 <- (sgolayfilt(pupLf$dia, p=3, n=31, m=1))*100+6
pupLf$dia_sm2 <- (sgolayfilt(pupLf$dia, p=3, n=31, m=2))*100+6


#Erkennung der Tiefpunkte der 2. Ableitung
vly <- ggplot(data = pupLf, aes(x=time, y=dia_sm2)) + 
  stat_valleys(span = 601, col = "green")
vly_data <- (layer_data(vly, i=1L)$xintercept)

#Darstellung der Tiefpunkte der 2. Ableitung
Derivative_test <- ggplot(data = pupLf, aes(x=time, y=dia_sm2, col="valley")) + 
  geom_line(data = pupLf, aes(x = time, y = dia_sm, col = "Diameter")) +
  geom_line(data = pupRf, aes(x = time, y = dia_sm, col = "Diameter")) +
    geom_line(aes(col = "2nd derivative"), alpha = 0.6) +
  stat_valleys(span = 601, col = "green")+
  geom_vline(xintercept = vly_data-0.1, lwd = 0.1, col="green")+
  geom_text(aes(label = paste("n=1, ", Code, ", Up", sep=""), x = 90, y= 8), size = 2.5)+
  theme_cowplot(font_size = 8, font_family = "sans")+
  scale_color_brewer(palette = "Set1", "Type")+
  ggtitle("Pupil-diameter data smoothed with 2nd derivative and valleys (-100ms)")+
  ylab("Pupil diameter / mm") +
  xlab("Time / seconds")+
  theme(legend.position = c(0.9,.1),legend.background = element_rect(fill="white", size = 0.5))+
  coord_cartesian(xlim = c(80, 200))

Derivative_test
ggsave("Pupil_Derivative_test.pdf", path = paste("../figures/", Code, sep=""), width=10, height = 5)
```


```
#Wahl eine Programmstartpunkts

vly_data <- tibble("index" = 1: length(vly_data), "time" = vly_data-0.1, "dif" = c(vly_data[1], diff(vly_data)))
kable(vly_data[1:30,], "pandoc", caption = "Time of 2nd derivative valleys (-100ms) first 30 rows", align = "c")# Tiefpunkte mit Zeitstempel
```


```
Registered S3 methods overwritten by 'htmltools':
  method               from         
  print.html           tools:rstudio
  print.shiny.tag      tools:rstudio
  print.shiny.tag.list tools:rstudio
```


Time of 2nd derivative valleys (-100ms) first 30 rows

| index | time | dif |
| --- | --- | --- |
| 1 | 7.805 | 7.905 |
| 2 | 18.505 | 10.700 |
| 3 | 27.272 | 8.767 |
| 4 | 37.522 | 10.250 |
| 5 | 55.572 | 18.050 |
| 6 | 64.972 | 9.400 |
| 7 | 78.455 | 13.483 |
| 8 | 97.338 | 18.883 |
| 9 | 110.822 | 13.484 |
| 10 | 120.955 | 10.133 |
| 11 | 136.822 | 15.867 |
| 12 | 143.622 | 6.800 |
| 13 | 161.205 | 17.583 |
| 14 | 170.455 | 9.250 |
| 15 | 178.055 | 7.600 |
| 16 | 186.105 | 8.050 |
| 17 | 197.555 | 11.450 |
| 18 | 203.922 | 6.367 |
| 19 | 210.972 | 7.050 |
| 20 | 223.638 | 12.666 |
| 21 | 231.722 | 8.084 |
| 22 | 246.572 | 14.850 |
| 23 | 254.388 | 7.816 |
| 24 | 267.388 | 13.000 |
| 25 | 282.672 | 15.284 |
| 26 | 293.105 | 10.433 |
| 27 | 307.455 | 14.350 |
| 28 | 313.722 | 6.267 |
| 29 | 324.405 | 10.683 |
| 30 | 329.788 | 5.383 |


```
sequ_per <- 15.144 #Sequenz Periode -> Ermittlung aus Video mit Geräuschanalyse

#Welcher index ist der Startzeitpunkt?
i <- 9 # Hier muss man auf Basis der Grafik und der Tabelle den Startzeitpunkt angeben

#Zeitkorrektur notwendig?
cor <- 1.325


pupLf$time_c <- pupLf$time - vly_data$time[vly_data$index == i] + sequ_per*cor
pupRf$time_c <- pupRf$time - vly_data$time[vly_data$index == i] + sequ_per*cor

#Ergänzung der Wellenlängen 

wl_sec <- tibble(
  "wl" = prog$Peak_4, 
  "wl_b" = seq(from = 0, to = (length(prog$Peak_4)-1)*sequ_per, by = sequ_per),
  "wl_e" = seq(from = sequ_per, to = length(prog$Peak_4)*sequ_per, by = sequ_per)
  )

tot_per <- length(prog$Peak_4)*sequ_per

sequ <- seq(from=0, to = tot_per, by = sequ_per)
wl <- prog$Peak_4
prog$index <- 1:length(prog$Peak_4)

pupLf$wl_step <- as.numeric(cut(pupLf$time_c,
               breaks = sequ,
               include.lowest = T,
               right = F,
               labels = wl))

pupLf$wl_time <-  pupLf$time_c - (pupLf$wl_step-1)*sequ_per

pupLf$wl_step <- prog$Peak_4[pupLf$wl_step]


pupRf$wl_step <- as.numeric(cut(pupRf$time_c,
               breaks = sequ,
               include.lowest = T,
               right = F,
               labels = wl))

pupRf$wl_time <-  pupRf$time_c - (pupRf$wl_step - 1)*sequ_per

pupRf$wl_step <- prog$Peak_4[pupRf$wl_step]

#Darstellung der Daten mit neuem Start und den jeweiligen Wellenlängen

Timed_plot <- ggplot() + 
  geom_rect(data = wl_sec, aes(xmin= wl_b, xmax = wl_e, ymin = 2, ymax = 8, fill = wl))+
  geom_line(data = pupLf, aes(x = time_c, y = dia_sm, col = "Left")) +
  geom_line(data = pupRf, aes(x = time_c, y = dia_sm, col = "Right"), alpha = 0.6) +
  geom_text(aes(label = paste("n=1, ", Code, ", Up", sep=""), x = 50, y= 7.5), size = 2.5)+
  theme_cowplot(font_size = 8, font_family = "sans")+
  scale_color_manual(values = c("grey80", "black"))+
  scale_fill_gradientn(colors = c(rev(rainbow(53, start = 0, end = 0.80)), rev(rainbow(8, start = 0.94, end = 1))))+
  ggtitle("Pupil-diameter during different wavelength")+
  ylab("Pupil diameter / mm") +
  xlab("Time / seconds")+ 
  coord_cartesian(ylim = c(2,8))+
  theme(legend.position = c(0.85,.22),legend.background = element_rect(fill="white", size = 0.5))

Timed_plot
ggsave("Timed_Smooth.pdf", path =  paste("../figures/", Code, sep=""),limitsize=F, width=20, height = 5)
```


```
WL_plot <- ggplot() + 
  geom_line(data = pupLf, aes(x = as.factor(wl_step), y = dia_sm, col = "Left")) +
  geom_line(data = pupRf, aes(x = as.factor(wl_step), y = dia_sm, col = "Right"), alpha = 0.6) +
  geom_text(aes(label = paste("n=1, ", Code, ", Up", sep=""), x = 50, y= 8), size = 2.5)+
  theme_cowplot(font_size = 8, font_family = "sans")+
  scale_color_brewer(palette = "Set1", "Eye")+
  ggtitle("Pupil-diameter per wavelength")+
  ylab("Pupil diameter / mm") +
  xlab("wavelength / nm")+ 
  coord_cartesian(ylim = c(2,8))+
  theme(legend.position = c(0.9,.1),legend.background = element_rect(fill="white", size = 0.5))

WL_plot
ggsave("Wavelength_Plot.pdf", path =  paste("../figures/", Code, sep=""),limitsize=F, width=10, height = 5)
```


```
WL_plot2 <- ggplot() + 
  geom_line(data = pupLf, aes(x = wl_time, y = dia_sm, group = wl_step, col = wl_step)) +
  geom_text(aes(label = paste("n=1, ", Code, ", Up", sep=""), x = 0.5, y= 8), size = 2.5)+
  theme_cowplot(font_size = 8, font_family = "sans")+
  scale_color_gradientn(colors = c(rev(rainbow(53, start = 0, end = 0.80)), rev(rainbow(8, start = 0.94, end = 1))))+
  ggtitle("Pupil-diameter depending on wavelength")+
  ylab("Pupil diameter / mm") +
  xlab("Time / seconds")+ 
  coord_cartesian(ylim = c(2,8))+
  theme(legend.position = c(0.9,.1),legend.background = element_rect(fill="white", size = 0.5))

WL_plot2
ggsave("Wavelength_Plot2.pdf", path =  paste("../figures/", Code, sep=""),limitsize=F, width=10, height = 7.5)
```


```
WL_plot2log <- ggplot() + 
  geom_line(data = pupLf, aes(x = log10(wl_time), y = dia_sm, group = wl_step, col = wl_step)) +
  geom_text(aes(label = paste("n=1, ", Code, ", Up", sep=""), x = -1, y= 8), size = 2.5)+
  theme_cowplot(font_size = 8, font_family = "sans")+
  scale_color_gradientn(colors = c(rev(rainbow(53, start = 0, end = 0.80)), rev(rainbow(8, start = 0.94, end = 1))))+
  ggtitle("Pupil-diameter depending on wavelength")+
  ylab("Pupil diameter / mm") +
  xlab("Time / log10(seconds)")+ 
  coord_cartesian(ylim = c(2,8), xlim = c(-1.5, log10(15)))+
  theme(legend.position = c(0.9,.1),legend.background = element_rect(fill="white", size = 0.5))

WL_plot2log
ggsave("Wavelength_Plot2log.pdf", path =  paste("../figures/", Code, sep=""),limitsize=F, width=10, height = 7.5)
```


Im folgenden Abschnitt werden Mittelwerte für jede Sekunde des Lichtreizes gebildet. Diese werden normiert, bezogen auf die Differenz zwischen der maximalen und minimalen Pupillenweite.


```
# Zunächst wird die -Baseline-Größe bestimmt. Sie wird hier definiert als der Mittelwert der Pupille in den letzten 5 Sekunden der Dunkeladaptation
# In Fällen, in denen die Pupille während der Belichtung niedrigere Werte annimmt, kann auch die größte Pupillengröße während der Belichtung gewählt werden. Dies erfolgt automatisch anhand des größeren Werts

L_norm <- as.numeric(
                  ifelse(pupLf %>% dplyr::filter(time_c < 0 & time_c > -5)  %>% summarize(p_max = mean(dia_sm)) > 
                   pupLf %>% dplyr::filter(time_c > 0 & time_c < tot_per)  %>% summarize(p_max = max(dia_sm)), 
                 pupLf %>% dplyr::filter(time_c < 0 & time_c > -5)  %>% summarize(p_max = mean(dia_sm)), 
                 pupLf %>% dplyr::filter(time_c > 0 & time_c < tot_per)  %>% summarize(p_max = max(dia_sm))
                 ))

# Im nächsten Schritt werden die Sekundenmittelwerte für jede Wellenlänge gebildet, zudem der Mittelwert der letzten 5 Sekunden und die minimalen Pupillengrößen für die jeweiligen Zeitintervalle

L_val <- pupLf %>% group_by(wl_step, wl_time %/% 1) %>% summarize(d_abs = mean(dia_sm))
```


```
`summarise()` regrouping output by 'wl_step' (override with `.groups` argument)
```


```
L_val10 <- pupLf %>% dplyr::filter(wl_time <= 15 & wl_time >= 10)  %>% group_by(wl_step) %>% summarize(d_abs = mean(dia_sm))
```


```
`summarise()` ungrouping output (override with `.groups` argument)
```


```
names(L_val) <- c("wl", "time", "d_abs")
names(L_val10) <- c("wl", "d_abs")

temp <- L_val %>% dplyr::filter(wl != "NA") %>% group_by(time) %>% summarize(min = min(d_abs))
```


```
`summarise()` ungrouping output (override with `.groups` argument)
```


```
L_norm <- data_frame("Cond" = c("Dark", temp$time, "10_15"), "dia" = c(L_norm, temp$min, min(L_val10$d_abs)))
```


```
`data_frame()` is deprecated as of tibble 1.1.0.
Please use `tibble()` instead.
[90mThis warning is displayed once every 8 hours.[39m
[90mCall `lifecycle::last_warnings()` to see where this warning was generated.[39m
```


```
# Im folgenden Schritt werden relative Pupillenkonstriktionswerte gebildet, amp ist dabei die Konstriktionsamplitude bezogen auf die Baseline-Größe, amp2 auf die Spanne zwischen der Basline-Größe und der kleinsten, für diese Zeitspanne gemessenen Pupillengröße.

temp <- as.numeric(as.character((cut(L_val$time,
               breaks = 0:(sequ_per %/% 1 +1),
               include.lowest = T,
               right = F,
               labels = L_norm$dia[2:(length(L_norm$dia)-1)]))
)) 

# Hier wird zunächst eine temporäre Variable erzeugt, welche für jede Zeile in den Sekundenmittelwertdaten die korrespondierende minimale Pupillengröße angibt.

L_val$amp <- (L_norm$dia[L_norm$Cond == "Dark"] - L_val$d_abs)/L_norm$dia[L_norm$Cond == "Dark"]*100
L_val10$amp <- (L_norm$dia[L_norm$Cond == "Dark"] - L_val10$d_abs)/L_norm$dia[L_norm$Cond == "Dark"]*100

L_val$amp2 <- (L_norm$dia[L_norm$Cond == "Dark"] - L_val$d_abs)/(L_norm$dia[L_norm$Cond == "Dark"] - temp)*100
L_val10$amp2 <- (L_norm$dia[L_norm$Cond == "Dark"] - L_val10$d_abs)/(L_norm$dia[L_norm$Cond == "Dark"] - L_norm$dia[L_norm$Cond == "10_15"])*100

##Nun auch für das rechte Auge
# Zunächst wird die -Baseline-Größe bestimmt. Sie wird hier definiert als der Mittelwert der Pupille in den letzten 5 Sekunden der Dunkeladaptation
# In Fällen, in denen die Pupille während der Belichtung niedrigere Werte annimmt, kann auch die größte Pupillengröße während der Belichtung gewählt werden. Dies erfolgt automatisch anhand des größeren Werts

        
R_norm <- as.numeric(
                  ifelse(ifelse(is.na(pupRf %>% dplyr::filter(time_c < 0 & time_c > -5)  %>% summarize(p_max = mean(dia_sm))), 0, pupRf %>% dplyr::filter(time_c < 0 & time_c > -5)  %>% summarize(p_max = mean(dia_sm))) > 
                   pupRf %>% dplyr::filter(time_c > 0 & time_c < tot_per)  %>% summarize(p_max = max(dia_sm)), 
                 pupRf %>% dplyr::filter(time_c < 0 & time_c > -5)  %>% summarize(p_max = mean(dia_sm)), 
                 pupRf %>% dplyr::filter(time_c > 0 & time_c < tot_per)  %>% summarize(p_max = max(dia_sm))
                 ))

# Im nächsten Schritt werden die Sekundenmittelwerte für jede Wellenlänge gebildet, zudem der Mittelwert der letzten 5 Sekunden und die minimalen Pupillengrößen für die jeweiligen Zeitintervalle

R_val <- pupRf %>% group_by(wl_step, wl_time %/% 1) %>% summarize(d_abs = mean(dia_sm))
```


```
`summarise()` regrouping output by 'wl_step' (override with `.groups` argument)
```


```
R_val10 <- pupRf %>% dplyr::filter(wl_time <= 15 & wl_time >= 10)  %>% group_by(wl_step) %>% summarize(d_abs = mean(dia_sm))
```


```
`summarise()` ungrouping output (override with `.groups` argument)
```


```
names(R_val) <- c("wl", "time", "d_abs")
names(R_val10) <- c("wl", "d_abs")

temp <- R_val %>% dplyr::filter(wl != "NA") %>% group_by(time) %>% summarize(min = min(d_abs))
```


```
`summarise()` ungrouping output (override with `.groups` argument)
```


```
R_norm <- data_frame("Cond" = c("Dark", temp$time, "10_15"), "dia" = c(R_norm, temp$min, min(R_val10$d_abs)))


# Im folgenden Schritt werden relative Pupillenkonstriktionswerte gebildet, amp ist dabei die Konstriktionsamplitude bezogen auf die Baseline-Größe, amp2 auf die Spanne zwischen der Basline-Größe und der kleinsten, für diese Zeitspanne gemessenen Pupillengröße.

temp <- as.numeric(as.character((cut(R_val$time,
               breaks = 0:(sequ_per %/% 1 +1),
               include.lowest = T,
               right = F,
               labels = R_norm$dia[2:(length(R_norm$dia)-1)]))
)) # Hier wird zunächst eine temporäre Variable erzeugt, welche für jede Zeile in den Sekundenmittelwertdaten die korrespondierende minimale Pupillengröße angibt.

R_val$amp <- (R_norm$dia[R_norm$Cond == "Dark"] - R_val$d_abs)/R_norm$dia[R_norm$Cond == "Dark"]*100
R_val10$amp <- (R_norm$dia[R_norm$Cond == "Dark"] - R_val10$d_abs)/R_norm$dia[R_norm$Cond == "Dark"]*100

R_val$amp2 <- (R_norm$dia[R_norm$Cond == "Dark"] - R_val$d_abs)/(R_norm$dia[R_norm$Cond == "Dark"] - temp)*100
R_val10$amp2 <- (R_norm$dia[R_norm$Cond == "Dark"] - R_val10$d_abs)/(R_norm$dia[R_norm$Cond == "Dark"] - R_norm$dia[R_norm$Cond == "10_15"])*100


#Abschließend die grafische Darstellung
Sens <-  ggplot() +
  geom_line(data = L_val, aes(x=wl, y=amp2, group = time, col = "Left"), alpha = 0.25) +
  geom_line(data = R_val, aes(x=wl, y=amp2, group = time, col = "Right"), alpha = 0.25) +
  geom_line(data = L_val10, aes(y=amp2, x=wl, col = "Left"), lwd = 1)+
  geom_line(data = R_val10, aes(y=amp2, x=wl, col = "Right"), lwd = 1)+
  geom_text(aes(label = paste("n=1, ", Code, ", Up", sep=""), x = 410, y= 100), size = 2.5)+
  theme_cowplot(font_size = 8, font_family = "sans")+
  scale_color_brewer(palette = "Set1", "Eye")+
  ggtitle("Sensitivity depending on wavelength and time (1-15 per second averages in lighter tone)")+
  ylab("Sensitivity / %") +
  xlab("Wavelength / nm")+ 
  theme(legend.position = c(0.9,.2),legend.background = element_rect(fill="white", size = 0.5))

Sens
ggsave("Sensitivity.pdf", path =  paste("../figures/", Code, sep=""),limitsize=F, width=10, height = 6)
```


Schließlich erfolgt der Export der Mittelwerte für alle Wellenlängen, sowie für die Normierungsrandbedingungen


```
#Entscheidung für ein Auge, auf Basis der meisten Datenverfügbarkeit, sofern nicht manuell etwas angegeben wird.

export <- ifelse(length(pupLf$dia_sm) > length(pupRf$dia_sm), "L", "R")
#export <- "L"

paste("the pupil of the following eye is used for export: ", ifelse(export == "R", "Right", "Left"))
```


```
[1] "the pupil of the following eye is used for export:  Left"
```


```
#Erstellung der Exportdaten für die Sekundenmittelwerte, die 10-15 Sekundenmittelwerte, und für die Normierungsrandbedingungen

temp <- get(paste(export, "_norm", sep=""))
export_data3 <- data.frame(t(temp[-1]))
colnames(export_data3) <- temp$Cond
export_data3 <- tibble("Code" = Code, "Eye" = ifelse(export == "R", "Right", "Left"), "Date" = as.Date(pup$UTC[1]), "Time" = format(as.POSIXct(pup$UTC[1]), format = "%H:%M:%S"), "Dir" = "Up", export_data3)

export_data0 <- get(paste("pup", export, "f", sep=""))
export_data0$amp1 <- (export_data3$Dark[1] - export_data0$dia_sm)/export_data3$Dark[1]*100

export_data1 <- get(paste(export, "_val", sep=""))[-length(get(paste(export, "_val", sep=""))$wl),]
export_data1$index <- match(export_data1$wl, wl)

export_data2 <- get(paste(export, "_val10", sep=""))
export_data2$index <- match(export_data2$wl, wl)

write_csv(export_data0, paste("../data_output/", Code, "_full.csv", sep="") )
write_csv(export_data1, paste("../data_output/", Code, "_all.csv", sep="") )
write_csv(export_data2, paste("../data_output/", Code, "_10_15.csv", sep="") )
write_csv(export_data3, paste("../data_output/", Code, "_norm.csv", sep="") )
```


```
#---- Anfang Code-Option

#Alternativ, wenn mit dem Rechten Auge die Zeit erfasst wird.

#Bildung der 1. und 2. Ableitung. Lineare Transformation, damit die Überlagerung mit dem Pupillendurchmesser lesbar bleibt.

pupRf$dia_sm1 <- (sgolayfilt(pupRf$dia, p=3, n=31, m=1))*100+6
pupRf$dia_sm2 <- (sgolayfilt(pupRf$dia, p=3, n=31, m=2))*100+6


#Erkennung der Tiefpunkte der 2. Ableitung
vly <- ggplot(data = pupRf, aes(x=time, y=dia_sm2)) + 
   stat_valleys(span = 601, col = "green")
 vly_data <- (layer_data(vly, i=1L)$xintercept)

#Darstellung der Tiefpunkte der 2. Ableitung
 Derivative_test <- ggplot(data = pupRf, aes(x=time, y=dia_sm2, col="valley")) + 
   geom_line(data = pupRf, aes(x = time, y = dia_sm, col = "Diameter")) +
   geom_line(data = pupLf, aes(x = time, y = dia_sm, col = "Diameter")) +
     geom_line(aes(col = "2st derivative"), alpha = 0.6) +
   stat_valleys(span = 601, col = "green")+
   geom_vline(xintercept = vly_data-0.1, lwd = 0.1, col="green")+
   geom_text(aes(label = paste("n=1, ", Code, sep=""), x = 85, y= 8), size = 2.5)+
   theme_cowplot(font_size = 8, font_family = "sans")+
   scale_color_brewer(palette = "Set1", "Type")+
   ggtitle("Pupil-diameter data smoothed with 2nd derivative and valleys (-100ms)")+
   ylab("Pupil diameter / mm") +
   xlab("Time / seconds")+
   theme(legend.position = c(0.9,.1),legend.background = element_rect(fill="white", size = 0.5))+
   coord_cartesian(xlim = c(80, 200))

 Derivative_test
 ggsave("Pupil_Derivative_test.pdf", path = paste("../figures/", Code, sep=""), width=10, height = 5)

#Wahl eines Programmstartpunkts

vly_data <- tibble("index" = 1: length(vly_data), "time" = vly_data-0.1, "dif" = c(vly_data[1], diff(vly_data)))
kable(vly_data[1:30,], "pandoc", caption = "Time of 2nd derivative valleys (-100ms), first 30 rows", align = "c")# Tiefpunkte mit Zeitstempel


#---- Ende Code-Option
```


LS0tCnRpdGxlOiAiUiBQdXBpbCIKb3V0cHV0OgogIGh0bWxfbm90ZWJvb2s6IGRlZmF1bHQKICBodG1sX2RvY3VtZW50OgogICAgZGZfcHJpbnQ6IHBhZ2VkCiAgcGRmX2RvY3VtZW50OiBkZWZhdWx0Ci0tLQpNaXQgZGllc2VtIERva3VtZW50IHdlcmRlbiBhdXMgZGVuIERpa2FibGlzIFB1cGlsbGVucm9oZGF0ZW4gbWl0IGRlbSBIb2Noc2NodWxlLU3DvG5jaGVuIExpY2h0bGFib3IoRDExMyktU2V0dXAgYXVzd2VydHVuZ3Nmw6RoaWdlIFB1cGlsbGVuZGF0ZW4gZXh0cmFoaWVydC4gRGllIEF1c3dlcnR1bmcgaXN0IGfDvGx0aWcgZsO8ciBkYXMgIlByb2dyYW1tIDQiIGF1cyBkZW0gTGlnaHRkb21lLVN0ZXVlcnJlY2huZXIgdW5kIGbDvHIgZGllICJuZXVlIiBVbXJlY2hudW5nc21ldGhvZGUgdm9uIFBpeGVsbiB6dSBtbSwgYmVpIGRlciB2b3IgQXVmemVpY2hudW5nIGVpbiBSZWZlcmVuem1hw58gYXVmIEF1Z2VuZWJlbmUgaW0gS2FtZXJhYmlsZCBnZXplaWd0IHdpcmQuIERpZXNlcyBQcm9ncmFtbSBiZWdpbm50IG1pdCBlaW5lciBEdW5rZWxhZGFwYXRpb25zcGVyaW9kZSB2b24gOTAgU2VrdW5kZW4uIERhbmFjaCB3ZXJkZW4gaW4gY2EuIDE1IFNla3VuZGVuLVNjaHJpdHRlbiBkaWUgV2VsbGVubMOkbmdlbiB2b24gNDAwLTcwMCBubSBpbiA1IG5tIFNjaHJpdHRlbiBhYmdlZmFocmVuLiAKClVtIGRlbiBDb2RlIGbDvHIgZWluZSBuZXVlIFBlcnNvbiBhbnp1cGFzc2VuLCBzaW5kIGRpZSBmb2xnZW5kZW4gRWluZ3JpZmZlIG5vdHdlbmRpZy4gWmVpbGVuYW5nYWJlbiBiZXppZWhlbiBzaWNoIGF1ZiBkYXMgKi5SbWQtRmlsZToKCjEuIFplaWxlIDM3IC0+IEVpbmbDvGdlbiBkZXMga29ycmVrdGVuIERhdGVpbmFtZW5zIC8gUGZhZAoKMi4gWmVpbGUgNDUgLT4gVmVyc3VjaHNwZXJzb25lbmNvZGUgZWluZsO8Z2VuCgozLiBaZWlsZW4gMTE2LTEyNiAtPiBQcsO8ZnVuZywgb2IgZGllIEtyaXRlcmllbiB6dXIgTWVzc3dlcnRhdXNzb3J0aWVydW5nIGJlaSBaaXJrdWxhcml0w6R0LCBhYnNvbHV0ZW0gQWJzY2huaXR0LCB1bmQgZGVtIFNjaGx1c3NzY2huaXR0IGluIE9yZG51bmcgc2luZAoKNC4gWmVpbGVuIDIwOS0yMTIgLT4gTmFjaCBQcsO8ZnVuZywgb2IgZGllIFRhbC1Fcmtlbm51bmcgZnVua3Rpb25pZXJ0LCBBbmdhYmUgZGVzIGtvcnJla3RlbiBJbmRleCBmw7xyIGRlbiBSZWZlcmVuenplaXRwdW5rdC4gU29sbHRlIGRhcyBiZWltIGxpbmtlbiBBdWdlIG5pY2h0IG3DtmdsaWNoIHNlaW4sIG11c3MgZGVyIHVudGVyc3RlIENvZGUtQ2h1bmsgYXVzIGRlciBEYXRlaSBtYW51ZWxsIGhvY2gga29waWVydCB3ZXJkZW4uIFNvbGx0ZSBkZXIgZXJzdGUgQmVsZXVjaHR1bmdzemVpdHB1bmt0IG5pY2h0IGVya2FubnQgd2VyZGVuLCBrYW5uIGVpbiBzcMOkdGVyZXIgWmVpdHB1bmt0IGFuZ2VnZWJlbiB3ZXJkZW4gLSBpbiBkaWVzZW0gRmFsbCBtdXNzIHNwZXppZml6aWVydCB3ZXJkZW4sIHVtIHdpZSB2aWVsZSBTY2hyaXR0ZSBkZXIgUHJvZ3JhbW1zdGFydCB6dXLDvGNrbGllZ3QuCgo1LiBaZWlsZSA0MjYvNDI3IC0+IERpZSBBdXN3YWhsLCB3ZWxjaGVzIEF1Z2Ugc2NobHVzc2VuZGxpY2ggZsO8ciBkZW4gRGF0ZW5leHBvcnQgdmVyd2VuZGV0IHdpcmQsIHdpcmQgc3RhbmRhcmRtw6TDn2lnIGF1dG9tYXRpc2NoIHZvbGx6b2dlbiBhbmhhbmQgZGVyIFB1cGlsbGUgbWl0IGRlbSBtZWlzdGVuIERhdGVucHVua3RlbiBuYWNoIGRlciBCZXJlaW5pZ3VuZy4gU29sbHRlIGRpZXNlIEF1c3dhaGwgbmljaHQgaW4gT3JkbnVuZyBzZWluLCBtdXNzIGhpZXIgbWFudWVsbCAiUiIgb2RlciAiTCIgZWluZ2V0cmFnZW4gd2VyZGVuLgoKCkltIGZvbGdlbmRlbiBBYnNjaG5pdHQgd2VyZGVuIGFsbGUgbm90d2VuZGlnZW4gQmlibGlvdGhla2VuIGdlbGFkZW4sIHNvd2llIGRpZSBSb2hkYXRlbiBlaW5nZWxlc2VuLiBEZXIgQWJzY2huaXR0IGVuZGV0IG1pdCBlaW5lciBEYXJzdGVsbHVuZyBkZXIgUm9oZGF0ZW4gZGVyIFB1cGlsbGVuLCBzb3dpZSBkZXIgWmlya3VsYXJpw6R0LgoKYGBge3IgRGF0ZW4gZWlubGVzZW59CmxpYnJhcnkodGlkeXZlcnNlKQpsaWJyYXJ5KGFueXRpbWUpCmxpYnJhcnkoc2lnbmFsKQpsaWJyYXJ5KGdncGxvdDIpCmxpYnJhcnkoY293cGxvdCkKbGlicmFyeShnZ3BtaXNjKQpsaWJyYXJ5KGtuaXRyKQpsaWJyYXJ5KHJlYWR4bCkKCnNldHdkKGRpcm5hbWUocnN0dWRpb2FwaTo6Z2V0QWN0aXZlRG9jdW1lbnRDb250ZXh0KCkkcGF0aCkpICMgU2V0emVuIGRlcyBBcmJlaXRzdmVyemVpY2huaXNzZXMgYXVmIGdsZWljaGVzIFZlcnplaWNobmlzIHdpZSBTY3JpcHQgCgpwdXAgPC0gcmVhZC50YWJsZSgiLi4vZGF0YV9pbnB1dC9VcC9LRzExMDUwMl80LiBNZXNzdW5nIFByNCAxNy4wOC4yMDIwIDEzMTgwM19Dc3ZEYXRhLnR4dCIsIHNlcCA9ICJcdCIsIGhlYWRlciA9IFRSVUUsIHN0cmluZ3NBc0ZhY3RvcnM9RikgIyBFaW5sZXNlbiBkZXIgRGF0ZW4gUHVwaWxsZQpwcm9nIDwtIHJlYWQudGFibGUoIi4uL2RhdGFfaW5wdXQvcHJvZ3JhbV9kYXRhX2xkLmNzdiIsIGRlYyA9ICIsIiwgc2VwID0gIjsiLCBoZWFkZXIgPSBUUlVFLCBzdHJpbmdzQXNGYWN0b3JzPUYpICMgRWlubGVzZW4gZGVyIFByb2dyYW1tZGF0ZW4KY29yZiA8LSByZWFkX2V4Y2VsKCIuLi9VzIhiZXJzaWNodF9WUC54bHN4Iiwgc2hlZXQgPSAiVWViZXJzaWNodF9WUCIsIG5hPSJOQSIpICNsb2FkIGluIHRoZSBmaWxlIHdpdGggYWxsIGluZGl2aWR1YWwgY29kZXMgIyBFaW5sZXNlbiBkZXIgS29ycmVrdHVyZmFrdG9yZW4KCiNBdXN3w6RobGVuIGRlciBub3R3ZW5kaWdlbiBTcGFsdGVuIHVuZCBTcGFsdGVuIHVtYmVuZW5uZW4KI1VtcmVjaG5lbiBkZXMgVVRDLVplaXRzdGVtcGVscyBpbiBsb2thbGUgWmVpdAojQmVyZWNobnVuZyBkZXMgUHVwaWxsZW5kdXJjaG1lc3NlcnMgKG51ciBmw7xyIFJlZmVyZW56bWHDnyAtIERpZSBLb3JyZWt0dXJmYWt0b3JlbiBhbGxlciBQcm9iYW5kZW4gd2VyZGVuIGluIGVpbmVyIHNlcGFyYXRlbiBEYXRlaSBnZXNwZWljaGVydCkKCkNvZGUgPC0gIktHMTEwNTAyXzIiCgpkaXIuY3JlYXRlKHBhc3RlKCIuLi9maWd1cmVzLyIsIENvZGUsIHNlcD0iIikpCgpwdXAgPC0gc2VsZWN0KHB1cCwgcmVjX3RpbWUsIFVUQywgRGlrYWJsaXMuUHJvZmVzc2lvbmFsX0V5ZS5EYXRhX09yaWdpbmFsX0xlZnQuRXllX1B1cGlsLkFyZWEsIERpa2FibGlzLlByb2Zlc3Npb25hbF9FeWUuRGF0YV9PcmlnaW5hbF9MZWZ0LkV5ZV9QdXBpbC5IZWlnaHQsIERpa2FibGlzLlByb2Zlc3Npb25hbF9FeWUuRGF0YV9PcmlnaW5hbF9MZWZ0LkV5ZV9QdXBpbC5XaWR0aCwgRGlrYWJsaXMuUHJvZmVzc2lvbmFsX0V5ZS5EYXRhX09yaWdpbmFsX1JpZ2h0LkV5ZV9QdXBpbC5BcmVhLCBEaWthYmxpcy5Qcm9mZXNzaW9uYWxfRXllLkRhdGFfT3JpZ2luYWxfUmlnaHQuRXllX1B1cGlsLkhlaWdodCwgRGlrYWJsaXMuUHJvZmVzc2lvbmFsX0V5ZS5EYXRhX09yaWdpbmFsX1JpZ2h0LkV5ZV9QdXBpbC5XaWR0aCkgCgpwdXAgPC0gdGliYmxlKHB1cCkKCmNvbG5hbWVzKHB1cCkgPC0gYygidGltZSIsICJVVEMiLCAiTGEiLCAiTGgiLCAiTHciLCAiUmEiLCAiUmgiLCAiUnciKQoKcHVwJFVUQyA8LSBhbnl0aW1lKHB1cCRVVEMvMTAwMCkKCnB1cCA8LSBzZXBhcmF0ZShwdXAsIHRpbWUsIGMoImgiLCAibSIsICJzIiksIHNlcCA9ICI6IiwgY29udmVydCA9IFRSVUUpCnB1cCR0aW1lIDwtIHB1cCRzICsgcHVwJG0qNjAgKyBwdXAkaCo2MCo2MAoKY29yZl9MIDwtIGNvcmYkS29ycl9MaW5rc1tjb3JmJENvZGUgPT0gQ29kZV0KCmNvcmZfUiA8LSBjb3JmJEtvcnJfUmVjaHRzW2NvcmYkQ29kZSA9PSBDb2RlXQoKcHVwJExkIDwtIDIqc3FydChwdXAkTGEvcGkpL2NvcmZfTAoKcHVwJFJkIDwtIDIqc3FydChwdXAkUmEvcGkpL2NvcmZfUgoKI0F1ZnRlaWxlbiBpbiBQdXBpbGxlIHJlY2h0ZXMgdW5kIGxpbmtlcyBBdWdlCgpwdXBSIDwtIHNlbGVjdChwdXAsICJ0aW1lIiwgIlVUQyIsICJSZCIsICJSYSIsICJSaCIsICJSdyIpCnB1cFIgPC0gZHBseXI6OmZpbHRlcihwdXBSLCAhaXMubmEoUmEpKQpjb2xuYW1lcyhwdXBSKSA8LSBjKCJ0aW1lIiwgIlVUQyIsICJkaWEiLCAiYXJlYSIsICJoZWlnaHQiLCAid2lkdGgiKQoKcHVwTCA8LSBzZWxlY3QocHVwLCAidGltZSIsICJVVEMiLCAiTGQiLCAiTGEiLCAiTGgiLCAiTHciKQpwdXBMIDwtIGRwbHlyOjpmaWx0ZXIocHVwTCwgIWlzLm5hKExhKSkKY29sbmFtZXMocHVwTCkgPC0gYygidGltZSIsICJVVEMiLCAiZGlhIiwgImFyZWEiLCAiaGVpZ2h0IiwgIndpZHRoIikKCiNEYXJzdGVsbHVuZyBkZXIgRXJnZWJuaXNzZSBpbSBQbG90CiMgUm9oZGF0ZW4KUm9oX3Bsb3QgPC0gZ2dwbG90KCkgKyAKICBnZW9tX2xpbmUoZGF0YSA9IHB1cEwsIGFlcyh4ID0gdGltZSwgeSA9IGRpYSwgY29sID0gIkxlZnQiKSkgKwogIGdlb21fbGluZShkYXRhID0gcHVwUiwgYWVzKHggPSB0aW1lLCB5ID0gZGlhLCBjb2wgPSAiUmlnaHQiKSwgYWxwaGEgPSAwLjYpICsKICBnZW9tX3RleHQoYWVzKGxhYmVsID0gcGFzdGUoIm49MSwgIiwgQ29kZSwgIiwgVXAiLCBzZXA9IiIpLCB4ID0gNTAsIHk9IDgpLCBzaXplID0gMi41KSsKICB0aGVtZV9jb3dwbG90KGZvbnRfc2l6ZSA9IDgsIGZvbnRfZmFtaWx5ID0gInNhbnMiKSsKICBzY2FsZV9jb2xvcl9icmV3ZXIocGFsZXR0ZSA9ICJTZXQxIiwgIkV5ZSIpKwogIGdndGl0bGUoIlB1cGlsLWRpYW1ldGVyIGRhdGEgdW5jaGFuZ2VkIikrCiAgeWxhYigiUHVwaWwgZGlhbWV0ZXIgLyBtbSIpICsKICB4bGFiKCJUaW1lIC8gc2Vjb25kcyIpKwogIHRoZW1lKGxlZ2VuZC5wb3NpdGlvbiA9IGMoMC45LC4xKSxsZWdlbmQuYmFja2dyb3VuZCA9IGVsZW1lbnRfcmVjdChmaWxsPSJ3aGl0ZSIsIHNpemUgPSAwLjUpKQoKUm9oX3Bsb3QKZ2dzYXZlKCJQdXBpbF9SYXcucGRmIiwgcGF0aCA9IHBhc3RlKCIuLi9maWd1cmVzLyIsIENvZGUsIHNlcD0iIiksIHdpZHRoPTEwLCBoZWlnaHQgPSA1KQoKIyBaaXJrdWxhcml0w6R0ClJvaF9wbG90MiA8LSBnZ3Bsb3QoKSArIAogIGdlb21fbGluZShkYXRhID0gcHVwTCwgYWVzKHggPSB0aW1lLCB5ID0gd2lkdGgvaGVpZ2h0LCBjb2wgPSAiTGVmdCIpKSArCiAgZ2VvbV9saW5lKGRhdGEgPSBwdXBSLCBhZXMoeCA9IHRpbWUsIHkgPSB3aWR0aC9oZWlnaHQsIGNvbCA9ICJSaWdodCIpLCBhbHBoYSA9IDAuNikgKwogIGdlb21fdGV4dChhZXMobGFiZWwgPSBwYXN0ZSgibj0xLCAiLCBDb2RlLCAiLCBVcCIsIHNlcD0iIiksIHggPSA1MCwgeT0gMS4wMSksIHNpemUgPSAyLjUpKwogIHRoZW1lX2Nvd3Bsb3QoZm9udF9zaXplID0gOCwgZm9udF9mYW1pbHkgPSAic2FucyIpKwogIHNjYWxlX2NvbG9yX2JyZXdlcihwYWxldHRlID0gIlNldDEiLCAiRXllIikrCiAgZ2d0aXRsZSgiUHVwaWwtY2lyY3VsYXJpdHkiKSsKICB5bGFiKCJQdXBpbCBkaWFtZXRlciAvIG1tIikgKwogIHhsYWIoIlRpbWUgLyBzZWNvbmRzIikrCiAgdGhlbWUobGVnZW5kLnBvc2l0aW9uID0gYygwLjksLjEpLGxlZ2VuZC5iYWNrZ3JvdW5kID0gZWxlbWVudF9yZWN0KGZpbGw9IndoaXRlIiwgc2l6ZSA9IDAuNSkpCgpSb2hfcGxvdDIKZ2dzYXZlKCJQdXBpbF9SYXcyLnBkZiIsIHBhdGggPSBwYXN0ZSgiLi4vZmlndXJlcy8iLCBDb2RlLCBzZXA9IiIpLCB3aWR0aD0xMCwgaGVpZ2h0ID0gNSkKCgpgYGAKCgpJbSBrb21tZW5kZW4gQWJzY2huaXR0IHdlcmRlbiBBcnRlZmFrdGUgdW5kIHVucGxhdXNpYmxlIE1lc3N3ZXJ0ZSBlbnRmZXJudC4gWnVkZW0gd2lyZCBkaWUgS3VydmUgZ2VnbMOkdHRldC4KYGBge3IgRGF0ZW4gYXVmYmVyZWl0ZW59CiNFbnRmZXJudW5nIHZvbiBBdXNyZWlzc2VybiB1bmQgV2VydGVuIGJlaSBkZW5lbiBkaWUgUHVwaWxsZSBlaW5lIHppcmt1bGFyaXTDpHQgPCAwLDcgYXVmd2Vpc3QsIHp1ZGVtIHdpcmQgZGllIGxldHp0ZW4gMiBTZWt1bmRlbiBkZXIgTWVzc3VuZyBlbnRmZXJudAoKcHVwUmYgPC0gcHVwUlsxOihsZW5ndGgocHVwUiR0aW1lKS0xMjApLF0KcHVwUmYgPC0gZHBseXI6OmZpbHRlcihwdXBSZiwgZGlhID4gMikgIyBFbnRmZXJudW5nIHZvbiBXZXJ0ZW4ga2xlaW5lciBYIG1tCnB1cFJmIDwtIGRwbHlyOjpmaWx0ZXIocHVwUmYsIGRpYSA8IDkpICMgRW50ZmVybnVuZyB2b24gV2VydGVuIGdyw7bDn2VyIDggbW0KcHVwUmYgPC0gZHBseXI6OmZpbHRlcihwdXBSZiwgd2lkdGgvaGVpZ2h0ID4gMC43MCAmIHdpZHRoL2hlaWdodCA+IDAuNzApICMgRW50ZmVybnVuZyB2b24gV2VydGVuIGbDvHIgZGllIGRpZSBaaXJrdWxhcml0w6R0IGtsZWluZXIgYWxzIDAsNyBhdXNmw6RsbHQKcHVwUmYkZGlhX3NtIDwtIHNnb2xheWZpbHQocHVwUmYkZGlhLCBwPTMsIG49MzEpICMgR2zDpHR0dW5nIGRlciBLdXJ2ZSBtaXR0ZWxzIFNhdml0emt5LUdvbGF5IEZpbHRlcgoKcHVwTGYgPC0gcHVwTFsxOihsZW5ndGgocHVwTCR0aW1lKS0xMjApLF0KcHVwTGYgPC0gZHBseXI6OmZpbHRlcihwdXBMZiwgZGlhID4gMikgIyBFbnRmZXJudW5nIHZvbiBXZXJ0ZW4ga2xlaW5lciBYIG1tCnB1cExmIDwtIGRwbHlyOjpmaWx0ZXIocHVwTGYsIGRpYSA8IDkpICMgRW50ZmVybnVuZyB2b24gV2VydGVuIGdyw7bDn2VyIDggbW0KcHVwTGYgPC0gZHBseXI6OmZpbHRlcihwdXBMZiwgd2lkdGgvaGVpZ2h0ID4gMC43MCAmIHdpZHRoL2hlaWdodCA+IDAuNzApICMgRW50ZmVybnVuZyB2b24gV2VydGVuIGbDvHIgZGllIGRpZSBaaXJrdWxhcml0w6R0IGtsZWluZXIgYWxzIDAsNyBhdXNmw6RsbHQKcHVwTGYkZGlhX3NtIDwtIHNnb2xheWZpbHQocHVwTGYkZGlhLCBwPTMsIG49MzEpICMgR2zDpHR0dW5nIGRlciBLdXJ2ZSBtaXR0ZWxzIFNhdml0emt5LUdvbGF5IEZpbHRlcgoKU21vb3RoX3Bsb3RfdGVzdCA8LSBnZ3Bsb3QoKSArIAogIGdlb21fbGluZShkYXRhID0gcHVwTGYsIGFlcyh4ID0gdGltZSwgeSA9IGRpYSwgY29sID0gInVuc21vb3RoZWQiKSkgKwogIGdlb21fbGluZShkYXRhID0gcHVwTGYsIGFlcyh4ID0gdGltZSwgeSA9IGRpYV9zbSwgY29sID0gInNnIG49MzEiKSwgYWxwaGEgPSAwLjYpICsKICAgIGdlb21fbGluZShkYXRhID0gcHVwTGYsIGFlcyh4ID0gdGltZSwgeSA9IHNnb2xheWZpbHQoZGlhLCBwPTMsIG49NjEpLCBjb2wgPSAic2cgbj02MSIpLCBhbHBoYSA9IDAuNikgKwogICAgZ2VvbV9saW5lKGRhdGEgPSBwdXBMZiwgYWVzKHggPSB0aW1lLCB5ID0gc2dvbGF5ZmlsdChkaWEsIHA9Mywgbj05MSksIGNvbCA9ICJzZyBuPTkxIiksIGFscGhhID0gMC42KSArCiAgZ2VvbV90ZXh0KGFlcyhsYWJlbCA9IHBhc3RlKCJuPTEsICIsIENvZGUsICIsIFVwIiwgc2VwPSIiKSwgeCA9IDUwLCB5PSA4KSwgc2l6ZSA9IDIuNSkrCiAgdGhlbWVfY293cGxvdChmb250X3NpemUgPSA4LCBmb250X2ZhbWlseSA9ICJzYW5zIikrCiAgc2NhbGVfY29sb3JfYnJld2VyKHBhbGV0dGUgPSAiU2V0MSIsICJFeWUiKSsKICBnZ3RpdGxlKCJQdXBpbC1kaWFtZXRlciBkYXRhIHNtb290aGVkLCB0ZXN0IHNldHRpbmdzIikrCiAgeWxhYigiUHVwaWwgZGlhbWV0ZXIgLyBtbSIpICsKICB4bGFiKCJUaW1lIC8gc2Vjb25kcyIpKwogIHRoZW1lKGxlZ2VuZC5wb3NpdGlvbiA9IGMoMC45LC4xKSxsZWdlbmQuYmFja2dyb3VuZCA9IGVsZW1lbnRfcmVjdChmaWxsPSJ3aGl0ZSIsIHNpemUgPSAwLjUpKSsKICBjb29yZF9jYXJ0ZXNpYW4oeGxpbSA9IGMoMjUwLCAyNzUpLCB5bGltID0gYygyLDgpKQoKU21vb3RoX3Bsb3RfdGVzdApnZ3NhdmUoIlB1cGlsX1Ntb290aF90ZXN0LnBkZiIsIHBhdGggPSBwYXN0ZSgiLi4vZmlndXJlcy8iLCBDb2RlLCBzZXA9IiIpLCB3aWR0aD0xMCwgaGVpZ2h0ID0gNSkKClNtb290aF9wbG90IDwtIGdncGxvdCgpICsgCiAgZ2VvbV9saW5lKGRhdGEgPSBwdXBMZiwgYWVzKHggPSB0aW1lLCB5ID0gZGlhX3NtLCBjb2wgPSAiTGVmdCIpKSArCiAgZ2VvbV9saW5lKGRhdGEgPSBwdXBSZiwgYWVzKHggPSB0aW1lLCB5ID0gZGlhX3NtLCBjb2wgPSAiUmlnaHQiKSwgYWxwaGEgPSAwLjYpICsKICBnZW9tX3RleHQoYWVzKGxhYmVsID0gcGFzdGUoIm49MSwgIiwgQ29kZSwgIiwgVXAiLCBzZXA9IiIpLCB4ID0gNTAsIHk9IDgpLCBzaXplID0gMi41KSsKICB0aGVtZV9jb3dwbG90KGZvbnRfc2l6ZSA9IDgsIGZvbnRfZmFtaWx5ID0gInNhbnMiKSsKICBzY2FsZV9jb2xvcl9icmV3ZXIocGFsZXR0ZSA9ICJTZXQxIiwgIkV5ZSIpKwogIGdndGl0bGUoIlB1cGlsLWRpYW1ldGVyIGRhdGEgc21vb3RoZWQiKSsKICB5bGFiKCJQdXBpbCBkaWFtZXRlciAvIG1tIikgKwogIHhsYWIoIlRpbWUgLyBzZWNvbmRzIikrIAogIGNvb3JkX2NhcnRlc2lhbih5bGltID0gYygyLDgpKSsKICB0aGVtZShsZWdlbmQucG9zaXRpb24gPSBjKDAuOSwuMSksbGVnZW5kLmJhY2tncm91bmQgPSBlbGVtZW50X3JlY3QoZmlsbD0id2hpdGUiLCBzaXplID0gMC41KSkKClNtb290aF9wbG90Cmdnc2F2ZSgiUHVwaWxfU21vb3RoLnBkZiIsIHBhdGggPSAgcGFzdGUoIi4uL2ZpZ3VyZXMvIiwgQ29kZSwgc2VwPSIiKSwgd2lkdGg9MTAsIGhlaWdodCA9IDUpCgoKYGBgCgoKSW0gbsOkY2hzdGVuIEFic2Nobml0dCBlcmZvbGd0IGRpZSBadW9yZG51bmcgZWluemVsbmVyIFplaXRhYnNjaG5pdHRlIHp1IGRlbiBXZWxsZW5sw6RuZ2VuLgpgYGB7ciBXZWxsZW5sw6RuZ2VuIHp1b3JkbmVufQoKI0JpbGR1bmcgZGVyIDEuIHVuZCAyLiBBYmxlaXR1bmcuIExpbmVhcmUgVHJhbnNmb3JtYXRpb24sIGRhbWl0IGRpZSDDnGJlcmxhZ2VydW5nIG1pdCBkZW0gUHVwaWxsZW5kdXJjaG1lc3NlciBsZXNiYXIgYmxlaWJ0LgoKcHVwTGYkZGlhX3NtMSA8LSAoc2dvbGF5ZmlsdChwdXBMZiRkaWEsIHA9Mywgbj0zMSwgbT0xKSkqMTAwKzYKcHVwTGYkZGlhX3NtMiA8LSAoc2dvbGF5ZmlsdChwdXBMZiRkaWEsIHA9Mywgbj0zMSwgbT0yKSkqMTAwKzYKCgojRXJrZW5udW5nIGRlciBUaWVmcHVua3RlIGRlciAyLiBBYmxlaXR1bmcKdmx5IDwtIGdncGxvdChkYXRhID0gcHVwTGYsIGFlcyh4PXRpbWUsIHk9ZGlhX3NtMikpICsgCiAgc3RhdF92YWxsZXlzKHNwYW4gPSA2MDEsIGNvbCA9ICJncmVlbiIpCnZseV9kYXRhIDwtIChsYXllcl9kYXRhKHZseSwgaT0xTCkkeGludGVyY2VwdCkKCiNEYXJzdGVsbHVuZyBkZXIgVGllZnB1bmt0ZSBkZXIgMi4gQWJsZWl0dW5nCkRlcml2YXRpdmVfdGVzdCA8LSBnZ3Bsb3QoZGF0YSA9IHB1cExmLCBhZXMoeD10aW1lLCB5PWRpYV9zbTIsIGNvbD0idmFsbGV5IikpICsgCiAgZ2VvbV9saW5lKGRhdGEgPSBwdXBMZiwgYWVzKHggPSB0aW1lLCB5ID0gZGlhX3NtLCBjb2wgPSAiRGlhbWV0ZXIiKSkgKwogIGdlb21fbGluZShkYXRhID0gcHVwUmYsIGFlcyh4ID0gdGltZSwgeSA9IGRpYV9zbSwgY29sID0gIkRpYW1ldGVyIikpICsKICAgIGdlb21fbGluZShhZXMoY29sID0gIjJuZCBkZXJpdmF0aXZlIiksIGFscGhhID0gMC42KSArCiAgc3RhdF92YWxsZXlzKHNwYW4gPSA2MDEsIGNvbCA9ICJncmVlbiIpKwogIGdlb21fdmxpbmUoeGludGVyY2VwdCA9IHZseV9kYXRhLTAuMSwgbHdkID0gMC4xLCBjb2w9ImdyZWVuIikrCiAgZ2VvbV90ZXh0KGFlcyhsYWJlbCA9IHBhc3RlKCJuPTEsICIsIENvZGUsICIsIFVwIiwgc2VwPSIiKSwgeCA9IDkwLCB5PSA4KSwgc2l6ZSA9IDIuNSkrCiAgdGhlbWVfY293cGxvdChmb250X3NpemUgPSA4LCBmb250X2ZhbWlseSA9ICJzYW5zIikrCiAgc2NhbGVfY29sb3JfYnJld2VyKHBhbGV0dGUgPSAiU2V0MSIsICJUeXBlIikrCiAgZ2d0aXRsZSgiUHVwaWwtZGlhbWV0ZXIgZGF0YSBzbW9vdGhlZCB3aXRoIDJuZCBkZXJpdmF0aXZlIGFuZCB2YWxsZXlzICgtMTAwbXMpIikrCiAgeWxhYigiUHVwaWwgZGlhbWV0ZXIgLyBtbSIpICsKICB4bGFiKCJUaW1lIC8gc2Vjb25kcyIpKwogIHRoZW1lKGxlZ2VuZC5wb3NpdGlvbiA9IGMoMC45LC4xKSxsZWdlbmQuYmFja2dyb3VuZCA9IGVsZW1lbnRfcmVjdChmaWxsPSJ3aGl0ZSIsIHNpemUgPSAwLjUpKSsKICBjb29yZF9jYXJ0ZXNpYW4oeGxpbSA9IGMoODAsIDIwMCkpCgpEZXJpdmF0aXZlX3Rlc3QKZ2dzYXZlKCJQdXBpbF9EZXJpdmF0aXZlX3Rlc3QucGRmIiwgcGF0aCA9IHBhc3RlKCIuLi9maWd1cmVzLyIsIENvZGUsIHNlcD0iIiksIHdpZHRoPTEwLCBoZWlnaHQgPSA1KQoKI1dhaGwgZWluZSBQcm9ncmFtbXN0YXJ0cHVua3RzCgp2bHlfZGF0YSA8LSB0aWJibGUoImluZGV4IiA9IDE6IGxlbmd0aCh2bHlfZGF0YSksICJ0aW1lIiA9IHZseV9kYXRhLTAuMSwgImRpZiIgPSBjKHZseV9kYXRhWzFdLCBkaWZmKHZseV9kYXRhKSkpCmthYmxlKHZseV9kYXRhWzE6MzAsXSwgInBhbmRvYyIsIGNhcHRpb24gPSAiVGltZSBvZiAybmQgZGVyaXZhdGl2ZSB2YWxsZXlzICgtMTAwbXMpIGZpcnN0IDMwIHJvd3MiLCBhbGlnbiA9ICJjIikjIFRpZWZwdW5rdGUgbWl0IFplaXRzdGVtcGVsCgpgYGAKCmBgYHtyIFdlbGxlbmzDpG5nZW4genVvcmRuZW4yfQoKc2VxdV9wZXIgPC0gMTUuMTQ0ICNTZXF1ZW56IFBlcmlvZGUgLT4gRXJtaXR0bHVuZyBhdXMgVmlkZW8gbWl0IEdlcsOkdXNjaGFuYWx5c2UKCiNXZWxjaGVyIGluZGV4IGlzdCBkZXIgU3RhcnR6ZWl0cHVua3Q/CmkgPC0gOSAjIEhpZXIgbXVzcyBtYW4gYXVmIEJhc2lzIGRlciBHcmFmaWsgdW5kIGRlciBUYWJlbGxlIGRlbiBTdGFydHplaXRwdW5rdCBhbmdlYmVuCgojWmVpdGtvcnJla3R1ciBub3R3ZW5kaWc/CmNvciA8LSAxLjMyNQoKCnB1cExmJHRpbWVfYyA8LSBwdXBMZiR0aW1lIC0gdmx5X2RhdGEkdGltZVt2bHlfZGF0YSRpbmRleCA9PSBpXSArIHNlcXVfcGVyKmNvcgpwdXBSZiR0aW1lX2MgPC0gcHVwUmYkdGltZSAtIHZseV9kYXRhJHRpbWVbdmx5X2RhdGEkaW5kZXggPT0gaV0gKyBzZXF1X3Blcipjb3IKCiNFcmfDpG56dW5nIGRlciBXZWxsZW5sw6RuZ2VuIAoKd2xfc2VjIDwtIHRpYmJsZSgKICAid2wiID0gcHJvZyRQZWFrXzQsIAogICJ3bF9iIiA9IHNlcShmcm9tID0gMCwgdG8gPSAobGVuZ3RoKHByb2ckUGVha180KS0xKSpzZXF1X3BlciwgYnkgPSBzZXF1X3BlciksCiAgIndsX2UiID0gc2VxKGZyb20gPSBzZXF1X3BlciwgdG8gPSBsZW5ndGgocHJvZyRQZWFrXzQpKnNlcXVfcGVyLCBieSA9IHNlcXVfcGVyKQogICkKCnRvdF9wZXIgPC0gbGVuZ3RoKHByb2ckUGVha180KSpzZXF1X3BlcgoKc2VxdSA8LSBzZXEoZnJvbT0wLCB0byA9IHRvdF9wZXIsIGJ5ID0gc2VxdV9wZXIpCndsIDwtIHByb2ckUGVha180CnByb2ckaW5kZXggPC0gMTpsZW5ndGgocHJvZyRQZWFrXzQpCgpwdXBMZiR3bF9zdGVwIDwtIGFzLm51bWVyaWMoY3V0KHB1cExmJHRpbWVfYywKICAgICAgICAgICAgICAgYnJlYWtzID0gc2VxdSwKICAgICAgICAgICAgICAgaW5jbHVkZS5sb3dlc3QgPSBULAogICAgICAgICAgICAgICByaWdodCA9IEYsCiAgICAgICAgICAgICAgIGxhYmVscyA9IHdsKSkKCnB1cExmJHdsX3RpbWUgPC0gIHB1cExmJHRpbWVfYyAtIChwdXBMZiR3bF9zdGVwLTEpKnNlcXVfcGVyCgpwdXBMZiR3bF9zdGVwIDwtIHByb2ckUGVha180W3B1cExmJHdsX3N0ZXBdCgoKcHVwUmYkd2xfc3RlcCA8LSBhcy5udW1lcmljKGN1dChwdXBSZiR0aW1lX2MsCiAgICAgICAgICAgICAgIGJyZWFrcyA9IHNlcXUsCiAgICAgICAgICAgICAgIGluY2x1ZGUubG93ZXN0ID0gVCwKICAgICAgICAgICAgICAgcmlnaHQgPSBGLAogICAgICAgICAgICAgICBsYWJlbHMgPSB3bCkpCgpwdXBSZiR3bF90aW1lIDwtICBwdXBSZiR0aW1lX2MgLSAocHVwUmYkd2xfc3RlcCAtIDEpKnNlcXVfcGVyCgpwdXBSZiR3bF9zdGVwIDwtIHByb2ckUGVha180W3B1cFJmJHdsX3N0ZXBdCgojRGFyc3RlbGx1bmcgZGVyIERhdGVuIG1pdCBuZXVlbSBTdGFydCB1bmQgZGVuIGpld2VpbGlnZW4gV2VsbGVubMOkbmdlbgoKVGltZWRfcGxvdCA8LSBnZ3Bsb3QoKSArIAogIGdlb21fcmVjdChkYXRhID0gd2xfc2VjLCBhZXMoeG1pbj0gd2xfYiwgeG1heCA9IHdsX2UsIHltaW4gPSAyLCB5bWF4ID0gOCwgZmlsbCA9IHdsKSkrCiAgZ2VvbV9saW5lKGRhdGEgPSBwdXBMZiwgYWVzKHggPSB0aW1lX2MsIHkgPSBkaWFfc20sIGNvbCA9ICJMZWZ0IikpICsKICBnZW9tX2xpbmUoZGF0YSA9IHB1cFJmLCBhZXMoeCA9IHRpbWVfYywgeSA9IGRpYV9zbSwgY29sID0gIlJpZ2h0IiksIGFscGhhID0gMC42KSArCiAgZ2VvbV90ZXh0KGFlcyhsYWJlbCA9IHBhc3RlKCJuPTEsICIsIENvZGUsICIsIFVwIiwgc2VwPSIiKSwgeCA9IDUwLCB5PSA3LjUpLCBzaXplID0gMi41KSsKICB0aGVtZV9jb3dwbG90KGZvbnRfc2l6ZSA9IDgsIGZvbnRfZmFtaWx5ID0gInNhbnMiKSsKICBzY2FsZV9jb2xvcl9tYW51YWwodmFsdWVzID0gYygiZ3JleTgwIiwgImJsYWNrIikpKwogIHNjYWxlX2ZpbGxfZ3JhZGllbnRuKGNvbG9ycyA9IGMocmV2KHJhaW5ib3coNTMsIHN0YXJ0ID0gMCwgZW5kID0gMC44MCkpLCByZXYocmFpbmJvdyg4LCBzdGFydCA9IDAuOTQsIGVuZCA9IDEpKSkpKwogIGdndGl0bGUoIlB1cGlsLWRpYW1ldGVyIGR1cmluZyBkaWZmZXJlbnQgd2F2ZWxlbmd0aCIpKwogIHlsYWIoIlB1cGlsIGRpYW1ldGVyIC8gbW0iKSArCiAgeGxhYigiVGltZSAvIHNlY29uZHMiKSsgCiAgY29vcmRfY2FydGVzaWFuKHlsaW0gPSBjKDIsOCkpKwogIHRoZW1lKGxlZ2VuZC5wb3NpdGlvbiA9IGMoMC44NSwuMjIpLGxlZ2VuZC5iYWNrZ3JvdW5kID0gZWxlbWVudF9yZWN0KGZpbGw9IndoaXRlIiwgc2l6ZSA9IDAuNSkpCgpUaW1lZF9wbG90Cmdnc2F2ZSgiVGltZWRfU21vb3RoLnBkZiIsIHBhdGggPSAgcGFzdGUoIi4uL2ZpZ3VyZXMvIiwgQ29kZSwgc2VwPSIiKSxsaW1pdHNpemU9Riwgd2lkdGg9MjAsIGhlaWdodCA9IDUpCgpXTF9wbG90IDwtIGdncGxvdCgpICsgCiAgZ2VvbV9saW5lKGRhdGEgPSBwdXBMZiwgYWVzKHggPSBhcy5mYWN0b3Iod2xfc3RlcCksIHkgPSBkaWFfc20sIGNvbCA9ICJMZWZ0IikpICsKICBnZW9tX2xpbmUoZGF0YSA9IHB1cFJmLCBhZXMoeCA9IGFzLmZhY3Rvcih3bF9zdGVwKSwgeSA9IGRpYV9zbSwgY29sID0gIlJpZ2h0IiksIGFscGhhID0gMC42KSArCiAgZ2VvbV90ZXh0KGFlcyhsYWJlbCA9IHBhc3RlKCJuPTEsICIsIENvZGUsICIsIFVwIiwgc2VwPSIiKSwgeCA9IDUwLCB5PSA4KSwgc2l6ZSA9IDIuNSkrCiAgdGhlbWVfY293cGxvdChmb250X3NpemUgPSA4LCBmb250X2ZhbWlseSA9ICJzYW5zIikrCiAgc2NhbGVfY29sb3JfYnJld2VyKHBhbGV0dGUgPSAiU2V0MSIsICJFeWUiKSsKICBnZ3RpdGxlKCJQdXBpbC1kaWFtZXRlciBwZXIgd2F2ZWxlbmd0aCIpKwogIHlsYWIoIlB1cGlsIGRpYW1ldGVyIC8gbW0iKSArCiAgeGxhYigid2F2ZWxlbmd0aCAvIG5tIikrIAogIGNvb3JkX2NhcnRlc2lhbih5bGltID0gYygyLDgpKSsKICB0aGVtZShsZWdlbmQucG9zaXRpb24gPSBjKDAuOSwuMSksbGVnZW5kLmJhY2tncm91bmQgPSBlbGVtZW50X3JlY3QoZmlsbD0id2hpdGUiLCBzaXplID0gMC41KSkKCldMX3Bsb3QKZ2dzYXZlKCJXYXZlbGVuZ3RoX1Bsb3QucGRmIiwgcGF0aCA9ICBwYXN0ZSgiLi4vZmlndXJlcy8iLCBDb2RlLCBzZXA9IiIpLGxpbWl0c2l6ZT1GLCB3aWR0aD0xMCwgaGVpZ2h0ID0gNSkKCldMX3Bsb3QyIDwtIGdncGxvdCgpICsgCiAgZ2VvbV9saW5lKGRhdGEgPSBwdXBMZiwgYWVzKHggPSB3bF90aW1lLCB5ID0gZGlhX3NtLCBncm91cCA9IHdsX3N0ZXAsIGNvbCA9IHdsX3N0ZXApKSArCiAgZ2VvbV90ZXh0KGFlcyhsYWJlbCA9IHBhc3RlKCJuPTEsICIsIENvZGUsICIsIFVwIiwgc2VwPSIiKSwgeCA9IDAuNSwgeT0gOCksIHNpemUgPSAyLjUpKwogIHRoZW1lX2Nvd3Bsb3QoZm9udF9zaXplID0gOCwgZm9udF9mYW1pbHkgPSAic2FucyIpKwogIHNjYWxlX2NvbG9yX2dyYWRpZW50bihjb2xvcnMgPSBjKHJldihyYWluYm93KDUzLCBzdGFydCA9IDAsIGVuZCA9IDAuODApKSwgcmV2KHJhaW5ib3coOCwgc3RhcnQgPSAwLjk0LCBlbmQgPSAxKSkpKSsKICBnZ3RpdGxlKCJQdXBpbC1kaWFtZXRlciBkZXBlbmRpbmcgb24gd2F2ZWxlbmd0aCIpKwogIHlsYWIoIlB1cGlsIGRpYW1ldGVyIC8gbW0iKSArCiAgeGxhYigiVGltZSAvIHNlY29uZHMiKSsgCiAgY29vcmRfY2FydGVzaWFuKHlsaW0gPSBjKDIsOCkpKwogIHRoZW1lKGxlZ2VuZC5wb3NpdGlvbiA9IGMoMC45LC4xKSxsZWdlbmQuYmFja2dyb3VuZCA9IGVsZW1lbnRfcmVjdChmaWxsPSJ3aGl0ZSIsIHNpemUgPSAwLjUpKQoKV0xfcGxvdDIKZ2dzYXZlKCJXYXZlbGVuZ3RoX1Bsb3QyLnBkZiIsIHBhdGggPSAgcGFzdGUoIi4uL2ZpZ3VyZXMvIiwgQ29kZSwgc2VwPSIiKSxsaW1pdHNpemU9Riwgd2lkdGg9MTAsIGhlaWdodCA9IDcuNSkKCldMX3Bsb3QybG9nIDwtIGdncGxvdCgpICsgCiAgZ2VvbV9saW5lKGRhdGEgPSBwdXBMZiwgYWVzKHggPSBsb2cxMCh3bF90aW1lKSwgeSA9IGRpYV9zbSwgZ3JvdXAgPSB3bF9zdGVwLCBjb2wgPSB3bF9zdGVwKSkgKwogIGdlb21fdGV4dChhZXMobGFiZWwgPSBwYXN0ZSgibj0xLCAiLCBDb2RlLCAiLCBVcCIsIHNlcD0iIiksIHggPSAtMSwgeT0gOCksIHNpemUgPSAyLjUpKwogIHRoZW1lX2Nvd3Bsb3QoZm9udF9zaXplID0gOCwgZm9udF9mYW1pbHkgPSAic2FucyIpKwogIHNjYWxlX2NvbG9yX2dyYWRpZW50bihjb2xvcnMgPSBjKHJldihyYWluYm93KDUzLCBzdGFydCA9IDAsIGVuZCA9IDAuODApKSwgcmV2KHJhaW5ib3coOCwgc3RhcnQgPSAwLjk0LCBlbmQgPSAxKSkpKSsKICBnZ3RpdGxlKCJQdXBpbC1kaWFtZXRlciBkZXBlbmRpbmcgb24gd2F2ZWxlbmd0aCIpKwogIHlsYWIoIlB1cGlsIGRpYW1ldGVyIC8gbW0iKSArCiAgeGxhYigiVGltZSAvIGxvZzEwKHNlY29uZHMpIikrIAogIGNvb3JkX2NhcnRlc2lhbih5bGltID0gYygyLDgpLCB4bGltID0gYygtMS41LCBsb2cxMCgxNSkpKSsKICB0aGVtZShsZWdlbmQucG9zaXRpb24gPSBjKDAuOSwuMSksbGVnZW5kLmJhY2tncm91bmQgPSBlbGVtZW50X3JlY3QoZmlsbD0id2hpdGUiLCBzaXplID0gMC41KSkKCldMX3Bsb3QybG9nCmdnc2F2ZSgiV2F2ZWxlbmd0aF9QbG90MmxvZy5wZGYiLCBwYXRoID0gIHBhc3RlKCIuLi9maWd1cmVzLyIsIENvZGUsIHNlcD0iIiksbGltaXRzaXplPUYsIHdpZHRoPTEwLCBoZWlnaHQgPSA3LjUpCgpgYGAKCgpJbSBmb2xnZW5kZW4gQWJzY2huaXR0IHdlcmRlbiBNaXR0ZWx3ZXJ0ZSBmw7xyIGplZGUgU2VrdW5kZSBkZXMgTGljaHRyZWl6ZXMgZ2ViaWxkZXQuIERpZXNlIHdlcmRlbiBub3JtaWVydCwgYmV6b2dlbiBhdWYgZGllIERpZmZlcmVueiB6d2lzY2hlbiBkZXIgbWF4aW1hbGVuIHVuZCBtaW5pbWFsZW4gUHVwaWxsZW53ZWl0ZS4KYGBge3IgTWl0dGVsd2VydHNiaWxkdW5nfQojIFp1bsOkY2hzdCB3aXJkIGRpZSAtQmFzZWxpbmUtR3LDtsOfZSBiZXN0aW1tdC4gU2llIHdpcmQgaGllciBkZWZpbmllcnQgYWxzIGRlciBNaXR0ZWx3ZXJ0IGRlciBQdXBpbGxlIGluIGRlbiBsZXR6dGVuIDUgU2VrdW5kZW4gZGVyIER1bmtlbGFkYXB0YXRpb24KIyBJbiBGw6RsbGVuLCBpbiBkZW5lbiBkaWUgUHVwaWxsZSB3w6RocmVuZCBkZXIgQmVsaWNodHVuZyBuaWVkcmlnZXJlIFdlcnRlIGFubmltbXQsIGthbm4gYXVjaCBkaWUgZ3LDtsOfdGUgUHVwaWxsZW5ncsO2w59lIHfDpGhyZW5kIGRlciBCZWxpY2h0dW5nIGdld8OkaGx0IHdlcmRlbi4gRGllcyBlcmZvbGd0IGF1dG9tYXRpc2NoIGFuaGFuZCBkZXMgZ3LDtsOfZXJlbiBXZXJ0cwoKTF9ub3JtIDwtIGFzLm51bWVyaWMoCiAgICAgICAgICAgICAgICAgIGlmZWxzZShwdXBMZiAlPiUgZHBseXI6OmZpbHRlcih0aW1lX2MgPCAwICYgdGltZV9jID4gLTUpICAlPiUgc3VtbWFyaXplKHBfbWF4ID0gbWVhbihkaWFfc20pKSA+IAogICAgICAgICAgICAgICAgICAgcHVwTGYgJT4lIGRwbHlyOjpmaWx0ZXIodGltZV9jID4gMCAmIHRpbWVfYyA8IHRvdF9wZXIpICAlPiUgc3VtbWFyaXplKHBfbWF4ID0gbWF4KGRpYV9zbSkpLCAKICAgICAgICAgICAgICAgICBwdXBMZiAlPiUgZHBseXI6OmZpbHRlcih0aW1lX2MgPCAwICYgdGltZV9jID4gLTUpICAlPiUgc3VtbWFyaXplKHBfbWF4ID0gbWVhbihkaWFfc20pKSwgCiAgICAgICAgICAgICAgICAgcHVwTGYgJT4lIGRwbHlyOjpmaWx0ZXIodGltZV9jID4gMCAmIHRpbWVfYyA8IHRvdF9wZXIpICAlPiUgc3VtbWFyaXplKHBfbWF4ID0gbWF4KGRpYV9zbSkpCiAgICAgICAgICAgICAgICAgKSkKCiMgSW0gbsOkY2hzdGVuIFNjaHJpdHQgd2VyZGVuIGRpZSBTZWt1bmRlbm1pdHRlbHdlcnRlIGbDvHIgamVkZSBXZWxsZW5sw6RuZ2UgZ2ViaWxkZXQsIHp1ZGVtIGRlciBNaXR0ZWx3ZXJ0IGRlciBsZXR6dGVuIDUgU2VrdW5kZW4gdW5kIGRpZSBtaW5pbWFsZW4gUHVwaWxsZW5ncsO2w59lbiBmw7xyIGRpZSBqZXdlaWxpZ2VuIFplaXRpbnRlcnZhbGxlCgpMX3ZhbCA8LSBwdXBMZiAlPiUgZ3JvdXBfYnkod2xfc3RlcCwgd2xfdGltZSAlLyUgMSkgJT4lIHN1bW1hcml6ZShkX2FicyA9IG1lYW4oZGlhX3NtKSkKTF92YWwxMCA8LSBwdXBMZiAlPiUgZHBseXI6OmZpbHRlcih3bF90aW1lIDw9IDE1ICYgd2xfdGltZSA+PSAxMCkgICU+JSBncm91cF9ieSh3bF9zdGVwKSAlPiUgc3VtbWFyaXplKGRfYWJzID0gbWVhbihkaWFfc20pKQoKbmFtZXMoTF92YWwpIDwtIGMoIndsIiwgInRpbWUiLCAiZF9hYnMiKQpuYW1lcyhMX3ZhbDEwKSA8LSBjKCJ3bCIsICJkX2FicyIpCgp0ZW1wIDwtIExfdmFsICU+JSBkcGx5cjo6ZmlsdGVyKHdsICE9ICJOQSIpICU+JSBncm91cF9ieSh0aW1lKSAlPiUgc3VtbWFyaXplKG1pbiA9IG1pbihkX2FicykpCgpMX25vcm0gPC0gZGF0YV9mcmFtZSgiQ29uZCIgPSBjKCJEYXJrIiwgdGVtcCR0aW1lLCAiMTBfMTUiKSwgImRpYSIgPSBjKExfbm9ybSwgdGVtcCRtaW4sIG1pbihMX3ZhbDEwJGRfYWJzKSkpCgoKIyBJbSBmb2xnZW5kZW4gU2Nocml0dCB3ZXJkZW4gcmVsYXRpdmUgUHVwaWxsZW5rb25zdHJpa3Rpb25zd2VydGUgZ2ViaWxkZXQsIGFtcCBpc3QgZGFiZWkgZGllIEtvbnN0cmlrdGlvbnNhbXBsaXR1ZGUgYmV6b2dlbiBhdWYgZGllIEJhc2VsaW5lLUdyw7bDn2UsIGFtcDIgYXVmIGRpZSBTcGFubmUgendpc2NoZW4gZGVyIEJhc2xpbmUtR3LDtsOfZSB1bmQgZGVyIGtsZWluc3RlbiwgZsO8ciBkaWVzZSBaZWl0c3Bhbm5lIGdlbWVzc2VuZW4gUHVwaWxsZW5ncsO2w59lLgoKdGVtcCA8LSBhcy5udW1lcmljKGFzLmNoYXJhY3RlcigoY3V0KExfdmFsJHRpbWUsCiAgICAgICAgICAgICAgIGJyZWFrcyA9IDA6KHNlcXVfcGVyICUvJSAxICsxKSwKICAgICAgICAgICAgICAgaW5jbHVkZS5sb3dlc3QgPSBULAogICAgICAgICAgICAgICByaWdodCA9IEYsCiAgICAgICAgICAgICAgIGxhYmVscyA9IExfbm9ybSRkaWFbMjoobGVuZ3RoKExfbm9ybSRkaWEpLTEpXSkpCikpIAoKIyBIaWVyIHdpcmQgenVuw6RjaHN0IGVpbmUgdGVtcG9yw6RyZSBWYXJpYWJsZSBlcnpldWd0LCB3ZWxjaGUgZsO8ciBqZWRlIFplaWxlIGluIGRlbiBTZWt1bmRlbm1pdHRlbHdlcnRkYXRlbiBkaWUga29ycmVzcG9uZGllcmVuZGUgbWluaW1hbGUgUHVwaWxsZW5ncsO2w59lIGFuZ2lidC4KCkxfdmFsJGFtcCA8LSAoTF9ub3JtJGRpYVtMX25vcm0kQ29uZCA9PSAiRGFyayJdIC0gTF92YWwkZF9hYnMpL0xfbm9ybSRkaWFbTF9ub3JtJENvbmQgPT0gIkRhcmsiXSoxMDAKTF92YWwxMCRhbXAgPC0gKExfbm9ybSRkaWFbTF9ub3JtJENvbmQgPT0gIkRhcmsiXSAtIExfdmFsMTAkZF9hYnMpL0xfbm9ybSRkaWFbTF9ub3JtJENvbmQgPT0gIkRhcmsiXSoxMDAKCkxfdmFsJGFtcDIgPC0gKExfbm9ybSRkaWFbTF9ub3JtJENvbmQgPT0gIkRhcmsiXSAtIExfdmFsJGRfYWJzKS8oTF9ub3JtJGRpYVtMX25vcm0kQ29uZCA9PSAiRGFyayJdIC0gdGVtcCkqMTAwCkxfdmFsMTAkYW1wMiA8LSAoTF9ub3JtJGRpYVtMX25vcm0kQ29uZCA9PSAiRGFyayJdIC0gTF92YWwxMCRkX2FicykvKExfbm9ybSRkaWFbTF9ub3JtJENvbmQgPT0gIkRhcmsiXSAtIExfbm9ybSRkaWFbTF9ub3JtJENvbmQgPT0gIjEwXzE1Il0pKjEwMAoKIyNOdW4gYXVjaCBmw7xyIGRhcyByZWNodGUgQXVnZQojIFp1bsOkY2hzdCB3aXJkIGRpZSAtQmFzZWxpbmUtR3LDtsOfZSBiZXN0aW1tdC4gU2llIHdpcmQgaGllciBkZWZpbmllcnQgYWxzIGRlciBNaXR0ZWx3ZXJ0IGRlciBQdXBpbGxlIGluIGRlbiBsZXR6dGVuIDUgU2VrdW5kZW4gZGVyIER1bmtlbGFkYXB0YXRpb24KIyBJbiBGw6RsbGVuLCBpbiBkZW5lbiBkaWUgUHVwaWxsZSB3w6RocmVuZCBkZXIgQmVsaWNodHVuZyBuaWVkcmlnZXJlIFdlcnRlIGFubmltbXQsIGthbm4gYXVjaCBkaWUgZ3LDtsOfdGUgUHVwaWxsZW5ncsO2w59lIHfDpGhyZW5kIGRlciBCZWxpY2h0dW5nIGdld8OkaGx0IHdlcmRlbi4gRGllcyBlcmZvbGd0IGF1dG9tYXRpc2NoIGFuaGFuZCBkZXMgZ3LDtsOfZXJlbiBXZXJ0cwoKICAgICAgICAKUl9ub3JtIDwtIGFzLm51bWVyaWMoCiAgICAgICAgICAgICAgICAgIGlmZWxzZShpZmVsc2UoaXMubmEocHVwUmYgJT4lIGRwbHlyOjpmaWx0ZXIodGltZV9jIDwgMCAmIHRpbWVfYyA+IC01KSAgJT4lIHN1bW1hcml6ZShwX21heCA9IG1lYW4oZGlhX3NtKSkpLCAwLCBwdXBSZiAlPiUgZHBseXI6OmZpbHRlcih0aW1lX2MgPCAwICYgdGltZV9jID4gLTUpICAlPiUgc3VtbWFyaXplKHBfbWF4ID0gbWVhbihkaWFfc20pKSkgPiAKICAgICAgICAgICAgICAgICAgIHB1cFJmICU+JSBkcGx5cjo6ZmlsdGVyKHRpbWVfYyA+IDAgJiB0aW1lX2MgPCB0b3RfcGVyKSAgJT4lIHN1bW1hcml6ZShwX21heCA9IG1heChkaWFfc20pKSwgCiAgICAgICAgICAgICAgICAgcHVwUmYgJT4lIGRwbHlyOjpmaWx0ZXIodGltZV9jIDwgMCAmIHRpbWVfYyA+IC01KSAgJT4lIHN1bW1hcml6ZShwX21heCA9IG1lYW4oZGlhX3NtKSksIAogICAgICAgICAgICAgICAgIHB1cFJmICU+JSBkcGx5cjo6ZmlsdGVyKHRpbWVfYyA+IDAgJiB0aW1lX2MgPCB0b3RfcGVyKSAgJT4lIHN1bW1hcml6ZShwX21heCA9IG1heChkaWFfc20pKQogICAgICAgICAgICAgICAgICkpCgojIEltIG7DpGNoc3RlbiBTY2hyaXR0IHdlcmRlbiBkaWUgU2VrdW5kZW5taXR0ZWx3ZXJ0ZSBmw7xyIGplZGUgV2VsbGVubMOkbmdlIGdlYmlsZGV0LCB6dWRlbSBkZXIgTWl0dGVsd2VydCBkZXIgbGV0enRlbiA1IFNla3VuZGVuIHVuZCBkaWUgbWluaW1hbGVuIFB1cGlsbGVuZ3LDtsOfZW4gZsO8ciBkaWUgamV3ZWlsaWdlbiBaZWl0aW50ZXJ2YWxsZQoKUl92YWwgPC0gcHVwUmYgJT4lIGdyb3VwX2J5KHdsX3N0ZXAsIHdsX3RpbWUgJS8lIDEpICU+JSBzdW1tYXJpemUoZF9hYnMgPSBtZWFuKGRpYV9zbSkpClJfdmFsMTAgPC0gcHVwUmYgJT4lIGRwbHlyOjpmaWx0ZXIod2xfdGltZSA8PSAxNSAmIHdsX3RpbWUgPj0gMTApICAlPiUgZ3JvdXBfYnkod2xfc3RlcCkgJT4lIHN1bW1hcml6ZShkX2FicyA9IG1lYW4oZGlhX3NtKSkKCm5hbWVzKFJfdmFsKSA8LSBjKCJ3bCIsICJ0aW1lIiwgImRfYWJzIikKbmFtZXMoUl92YWwxMCkgPC0gYygid2wiLCAiZF9hYnMiKQoKdGVtcCA8LSBSX3ZhbCAlPiUgZHBseXI6OmZpbHRlcih3bCAhPSAiTkEiKSAlPiUgZ3JvdXBfYnkodGltZSkgJT4lIHN1bW1hcml6ZShtaW4gPSBtaW4oZF9hYnMpKQoKUl9ub3JtIDwtIGRhdGFfZnJhbWUoIkNvbmQiID0gYygiRGFyayIsIHRlbXAkdGltZSwgIjEwXzE1IiksICJkaWEiID0gYyhSX25vcm0sIHRlbXAkbWluLCBtaW4oUl92YWwxMCRkX2FicykpKQoKCgojIEltIGZvbGdlbmRlbiBTY2hyaXR0IHdlcmRlbiByZWxhdGl2ZSBQdXBpbGxlbmtvbnN0cmlrdGlvbnN3ZXJ0ZSBnZWJpbGRldCwgYW1wIGlzdCBkYWJlaSBkaWUgS29uc3RyaWt0aW9uc2FtcGxpdHVkZSBiZXpvZ2VuIGF1ZiBkaWUgQmFzZWxpbmUtR3LDtsOfZSwgYW1wMiBhdWYgZGllIFNwYW5uZSB6d2lzY2hlbiBkZXIgQmFzbGluZS1HcsO2w59lIHVuZCBkZXIga2xlaW5zdGVuLCBmw7xyIGRpZXNlIFplaXRzcGFubmUgZ2VtZXNzZW5lbiBQdXBpbGxlbmdyw7bDn2UuCgp0ZW1wIDwtIGFzLm51bWVyaWMoYXMuY2hhcmFjdGVyKChjdXQoUl92YWwkdGltZSwKICAgICAgICAgICAgICAgYnJlYWtzID0gMDooc2VxdV9wZXIgJS8lIDEgKzEpLAogICAgICAgICAgICAgICBpbmNsdWRlLmxvd2VzdCA9IFQsCiAgICAgICAgICAgICAgIHJpZ2h0ID0gRiwKICAgICAgICAgICAgICAgbGFiZWxzID0gUl9ub3JtJGRpYVsyOihsZW5ndGgoUl9ub3JtJGRpYSktMSldKSkKKSkgIyBIaWVyIHdpcmQgenVuw6RjaHN0IGVpbmUgdGVtcG9yw6RyZSBWYXJpYWJsZSBlcnpldWd0LCB3ZWxjaGUgZsO8ciBqZWRlIFplaWxlIGluIGRlbiBTZWt1bmRlbm1pdHRlbHdlcnRkYXRlbiBkaWUga29ycmVzcG9uZGllcmVuZGUgbWluaW1hbGUgUHVwaWxsZW5ncsO2w59lIGFuZ2lidC4KClJfdmFsJGFtcCA8LSAoUl9ub3JtJGRpYVtSX25vcm0kQ29uZCA9PSAiRGFyayJdIC0gUl92YWwkZF9hYnMpL1Jfbm9ybSRkaWFbUl9ub3JtJENvbmQgPT0gIkRhcmsiXSoxMDAKUl92YWwxMCRhbXAgPC0gKFJfbm9ybSRkaWFbUl9ub3JtJENvbmQgPT0gIkRhcmsiXSAtIFJfdmFsMTAkZF9hYnMpL1Jfbm9ybSRkaWFbUl9ub3JtJENvbmQgPT0gIkRhcmsiXSoxMDAKClJfdmFsJGFtcDIgPC0gKFJfbm9ybSRkaWFbUl9ub3JtJENvbmQgPT0gIkRhcmsiXSAtIFJfdmFsJGRfYWJzKS8oUl9ub3JtJGRpYVtSX25vcm0kQ29uZCA9PSAiRGFyayJdIC0gdGVtcCkqMTAwClJfdmFsMTAkYW1wMiA8LSAoUl9ub3JtJGRpYVtSX25vcm0kQ29uZCA9PSAiRGFyayJdIC0gUl92YWwxMCRkX2FicykvKFJfbm9ybSRkaWFbUl9ub3JtJENvbmQgPT0gIkRhcmsiXSAtIFJfbm9ybSRkaWFbUl9ub3JtJENvbmQgPT0gIjEwXzE1Il0pKjEwMAoKCiNBYnNjaGxpZcOfZW5kIGRpZSBncmFmaXNjaGUgRGFyc3RlbGx1bmcKU2VucyA8LSAgZ2dwbG90KCkgKwogIGdlb21fbGluZShkYXRhID0gTF92YWwsIGFlcyh4PXdsLCB5PWFtcDIsIGdyb3VwID0gdGltZSwgY29sID0gIkxlZnQiKSwgYWxwaGEgPSAwLjI1KSArCiAgZ2VvbV9saW5lKGRhdGEgPSBSX3ZhbCwgYWVzKHg9d2wsIHk9YW1wMiwgZ3JvdXAgPSB0aW1lLCBjb2wgPSAiUmlnaHQiKSwgYWxwaGEgPSAwLjI1KSArCiAgZ2VvbV9saW5lKGRhdGEgPSBMX3ZhbDEwLCBhZXMoeT1hbXAyLCB4PXdsLCBjb2wgPSAiTGVmdCIpLCBsd2QgPSAxKSsKICBnZW9tX2xpbmUoZGF0YSA9IFJfdmFsMTAsIGFlcyh5PWFtcDIsIHg9d2wsIGNvbCA9ICJSaWdodCIpLCBsd2QgPSAxKSsKICBnZW9tX3RleHQoYWVzKGxhYmVsID0gcGFzdGUoIm49MSwgIiwgQ29kZSwgIiwgVXAiLCBzZXA9IiIpLCB4ID0gNDEwLCB5PSAxMDApLCBzaXplID0gMi41KSsKICB0aGVtZV9jb3dwbG90KGZvbnRfc2l6ZSA9IDgsIGZvbnRfZmFtaWx5ID0gInNhbnMiKSsKICBzY2FsZV9jb2xvcl9icmV3ZXIocGFsZXR0ZSA9ICJTZXQxIiwgIkV5ZSIpKwogIGdndGl0bGUoIlNlbnNpdGl2aXR5IGRlcGVuZGluZyBvbiB3YXZlbGVuZ3RoIGFuZCB0aW1lICgxLTE1IHBlciBzZWNvbmQgYXZlcmFnZXMgaW4gbGlnaHRlciB0b25lKSIpKwogIHlsYWIoIlNlbnNpdGl2aXR5IC8gJSIpICsKICB4bGFiKCJXYXZlbGVuZ3RoIC8gbm0iKSsgCiAgdGhlbWUobGVnZW5kLnBvc2l0aW9uID0gYygwLjksLjIpLGxlZ2VuZC5iYWNrZ3JvdW5kID0gZWxlbWVudF9yZWN0KGZpbGw9IndoaXRlIiwgc2l6ZSA9IDAuNSkpCgpTZW5zCmdnc2F2ZSgiU2Vuc2l0aXZpdHkucGRmIiwgcGF0aCA9ICBwYXN0ZSgiLi4vZmlndXJlcy8iLCBDb2RlLCBzZXA9IiIpLGxpbWl0c2l6ZT1GLCB3aWR0aD0xMCwgaGVpZ2h0ID0gNikKCmBgYAoKClNjaGxpZcOfbGljaCBlcmZvbGd0IGRlciBFeHBvcnQgZGVyIE1pdHRlbHdlcnRlIGbDvHIgYWxsZSBXZWxsZW5sw6RuZ2VuLCBzb3dpZSBmw7xyIGRpZSBOb3JtaWVydW5nc3JhbmRiZWRpbmd1bmdlbgpgYGB7ciBFeHBvcnR9CiNFbnRzY2hlaWR1bmcgZsO8ciBlaW4gQXVnZSwgYXVmIEJhc2lzIGRlciBtZWlzdGVuIERhdGVudmVyZsO8Z2JhcmtlaXQsIHNvZmVybiBuaWNodCBtYW51ZWxsIGV0d2FzIGFuZ2VnZWJlbiB3aXJkLgoKZXhwb3J0IDwtIGlmZWxzZShsZW5ndGgocHVwTGYkZGlhX3NtKSA+IGxlbmd0aChwdXBSZiRkaWFfc20pLCAiTCIsICJSIikKI2V4cG9ydCA8LSAiTCIKCnBhc3RlKCJ0aGUgcHVwaWwgb2YgdGhlIGZvbGxvd2luZyBleWUgaXMgdXNlZCBmb3IgZXhwb3J0OiAiLCBpZmVsc2UoZXhwb3J0ID09ICJSIiwgIlJpZ2h0IiwgIkxlZnQiKSkKCiNFcnN0ZWxsdW5nIGRlciBFeHBvcnRkYXRlbiBmw7xyIGRpZSBTZWt1bmRlbm1pdHRlbHdlcnRlLCBkaWUgMTAtMTUgU2VrdW5kZW5taXR0ZWx3ZXJ0ZSwgdW5kIGbDvHIgZGllIE5vcm1pZXJ1bmdzcmFuZGJlZGluZ3VuZ2VuCgp0ZW1wIDwtIGdldChwYXN0ZShleHBvcnQsICJfbm9ybSIsIHNlcD0iIikpCmV4cG9ydF9kYXRhMyA8LSBkYXRhLmZyYW1lKHQodGVtcFstMV0pKQpjb2xuYW1lcyhleHBvcnRfZGF0YTMpIDwtIHRlbXAkQ29uZApleHBvcnRfZGF0YTMgPC0gdGliYmxlKCJDb2RlIiA9IENvZGUsICJFeWUiID0gaWZlbHNlKGV4cG9ydCA9PSAiUiIsICJSaWdodCIsICJMZWZ0IiksICJEYXRlIiA9IGFzLkRhdGUocHVwJFVUQ1sxXSksICJUaW1lIiA9IGZvcm1hdChhcy5QT1NJWGN0KHB1cCRVVENbMV0pLCBmb3JtYXQgPSAiJUg6JU06JVMiKSwgIkRpciIgPSAiVXAiLCBleHBvcnRfZGF0YTMpCgpleHBvcnRfZGF0YTAgPC0gZ2V0KHBhc3RlKCJwdXAiLCBleHBvcnQsICJmIiwgc2VwPSIiKSkKZXhwb3J0X2RhdGEwJGFtcDEgPC0gKGV4cG9ydF9kYXRhMyREYXJrWzFdIC0gZXhwb3J0X2RhdGEwJGRpYV9zbSkvZXhwb3J0X2RhdGEzJERhcmtbMV0qMTAwCgpleHBvcnRfZGF0YTEgPC0gZ2V0KHBhc3RlKGV4cG9ydCwgIl92YWwiLCBzZXA9IiIpKVstbGVuZ3RoKGdldChwYXN0ZShleHBvcnQsICJfdmFsIiwgc2VwPSIiKSkkd2wpLF0KZXhwb3J0X2RhdGExJGluZGV4IDwtIG1hdGNoKGV4cG9ydF9kYXRhMSR3bCwgd2wpCgpleHBvcnRfZGF0YTIgPC0gZ2V0KHBhc3RlKGV4cG9ydCwgIl92YWwxMCIsIHNlcD0iIikpCmV4cG9ydF9kYXRhMiRpbmRleCA8LSBtYXRjaChleHBvcnRfZGF0YTIkd2wsIHdsKQoKd3JpdGVfY3N2KGV4cG9ydF9kYXRhMCwgcGFzdGUoIi4uL2RhdGFfb3V0cHV0LyIsIENvZGUsICJfZnVsbC5jc3YiLCBzZXA9IiIpICkKd3JpdGVfY3N2KGV4cG9ydF9kYXRhMSwgcGFzdGUoIi4uL2RhdGFfb3V0cHV0LyIsIENvZGUsICJfYWxsLmNzdiIsIHNlcD0iIikgKQp3cml0ZV9jc3YoZXhwb3J0X2RhdGEyLCBwYXN0ZSgiLi4vZGF0YV9vdXRwdXQvIiwgQ29kZSwgIl8xMF8xNS5jc3YiLCBzZXA9IiIpICkKd3JpdGVfY3N2KGV4cG9ydF9kYXRhMywgcGFzdGUoIi4uL2RhdGFfb3V0cHV0LyIsIENvZGUsICJfbm9ybS5jc3YiLCBzZXA9IiIpICkKCmBgYAoKCmBgYHtyIE9wdGlvbiwgZXZhbCA9IEZ9CiMtLS0tIEFuZmFuZyBDb2RlLU9wdGlvbgoKI0FsdGVybmF0aXYsIHdlbm4gbWl0IGRlbSBSZWNodGVuIEF1Z2UgZGllIFplaXQgZXJmYXNzdCB3aXJkLgoKI0JpbGR1bmcgZGVyIDEuIHVuZCAyLiBBYmxlaXR1bmcuIExpbmVhcmUgVHJhbnNmb3JtYXRpb24sIGRhbWl0IGRpZSDDnGJlcmxhZ2VydW5nIG1pdCBkZW0gUHVwaWxsZW5kdXJjaG1lc3NlciBsZXNiYXIgYmxlaWJ0LgoKcHVwUmYkZGlhX3NtMSA8LSAoc2dvbGF5ZmlsdChwdXBSZiRkaWEsIHA9Mywgbj0zMSwgbT0xKSkqMTAwKzYKcHVwUmYkZGlhX3NtMiA8LSAoc2dvbGF5ZmlsdChwdXBSZiRkaWEsIHA9Mywgbj0zMSwgbT0yKSkqMTAwKzYKCgojRXJrZW5udW5nIGRlciBUaWVmcHVua3RlIGRlciAyLiBBYmxlaXR1bmcKdmx5IDwtIGdncGxvdChkYXRhID0gcHVwUmYsIGFlcyh4PXRpbWUsIHk9ZGlhX3NtMikpICsgCiAgIHN0YXRfdmFsbGV5cyhzcGFuID0gNjAxLCBjb2wgPSAiZ3JlZW4iKQogdmx5X2RhdGEgPC0gKGxheWVyX2RhdGEodmx5LCBpPTFMKSR4aW50ZXJjZXB0KQoKI0RhcnN0ZWxsdW5nIGRlciBUaWVmcHVua3RlIGRlciAyLiBBYmxlaXR1bmcKIERlcml2YXRpdmVfdGVzdCA8LSBnZ3Bsb3QoZGF0YSA9IHB1cFJmLCBhZXMoeD10aW1lLCB5PWRpYV9zbTIsIGNvbD0idmFsbGV5IikpICsgCiAgIGdlb21fbGluZShkYXRhID0gcHVwUmYsIGFlcyh4ID0gdGltZSwgeSA9IGRpYV9zbSwgY29sID0gIkRpYW1ldGVyIikpICsKICAgZ2VvbV9saW5lKGRhdGEgPSBwdXBMZiwgYWVzKHggPSB0aW1lLCB5ID0gZGlhX3NtLCBjb2wgPSAiRGlhbWV0ZXIiKSkgKwogICAgIGdlb21fbGluZShhZXMoY29sID0gIjJzdCBkZXJpdmF0aXZlIiksIGFscGhhID0gMC42KSArCiAgIHN0YXRfdmFsbGV5cyhzcGFuID0gNjAxLCBjb2wgPSAiZ3JlZW4iKSsKICAgZ2VvbV92bGluZSh4aW50ZXJjZXB0ID0gdmx5X2RhdGEtMC4xLCBsd2QgPSAwLjEsIGNvbD0iZ3JlZW4iKSsKICAgZ2VvbV90ZXh0KGFlcyhsYWJlbCA9IHBhc3RlKCJuPTEsICIsIENvZGUsIHNlcD0iIiksIHggPSA4NSwgeT0gOCksIHNpemUgPSAyLjUpKwogICB0aGVtZV9jb3dwbG90KGZvbnRfc2l6ZSA9IDgsIGZvbnRfZmFtaWx5ID0gInNhbnMiKSsKICAgc2NhbGVfY29sb3JfYnJld2VyKHBhbGV0dGUgPSAiU2V0MSIsICJUeXBlIikrCiAgIGdndGl0bGUoIlB1cGlsLWRpYW1ldGVyIGRhdGEgc21vb3RoZWQgd2l0aCAybmQgZGVyaXZhdGl2ZSBhbmQgdmFsbGV5cyAoLTEwMG1zKSIpKwogICB5bGFiKCJQdXBpbCBkaWFtZXRlciAvIG1tIikgKwogICB4bGFiKCJUaW1lIC8gc2Vjb25kcyIpKwogICB0aGVtZShsZWdlbmQucG9zaXRpb24gPSBjKDAuOSwuMSksbGVnZW5kLmJhY2tncm91bmQgPSBlbGVtZW50X3JlY3QoZmlsbD0id2hpdGUiLCBzaXplID0gMC41KSkrCiAgIGNvb3JkX2NhcnRlc2lhbih4bGltID0gYyg4MCwgMjAwKSkKCiBEZXJpdmF0aXZlX3Rlc3QKIGdnc2F2ZSgiUHVwaWxfRGVyaXZhdGl2ZV90ZXN0LnBkZiIsIHBhdGggPSBwYXN0ZSgiLi4vZmlndXJlcy8iLCBDb2RlLCBzZXA9IiIpLCB3aWR0aD0xMCwgaGVpZ2h0ID0gNSkKCiNXYWhsIGVpbmVzIFByb2dyYW1tc3RhcnRwdW5rdHMKCnZseV9kYXRhIDwtIHRpYmJsZSgiaW5kZXgiID0gMTogbGVuZ3RoKHZseV9kYXRhKSwgInRpbWUiID0gdmx5X2RhdGEtMC4xLCAiZGlmIiA9IGModmx5X2RhdGFbMV0sIGRpZmYodmx5X2RhdGEpKSkKa2FibGUodmx5X2RhdGFbMTozMCxdLCAicGFuZG9jIiwgY2FwdGlvbiA9ICJUaW1lIG9mIDJuZCBkZXJpdmF0aXZlIHZhbGxleXMgKC0xMDBtcyksIGZpcnN0IDMwIHJvd3MiLCBhbGlnbiA9ICJjIikjIFRpZWZwdW5rdGUgbWl0IFplaXRzdGVtcGVsCgoKIy0tLS0gRW5kZSBDb2RlLU9wdGlvbgpgYGAKCg==
