# Supplementary material for: Spectral dependency of the human pupillary light reflex. Influences of pre-adaptation and chronotype
Source: PLoS One. 2022 Jan 12;17(1):e0253030. doi: 10.1371/journal.pone.0253030 (PMC8754338; doi:10.1371/journal.pone.0253030)
Supplement: S3 File — Zip file containing several files necessary to replicate the statistical analysis and generation of graphics. Besides some external tables, an R-function file, and three pictures to mark the respective experiment in graphs, the zip file consists of nine R-Markdown scripts. One of these is used to set up the data prior to analysis. The necessary data can be downloaded from the Open Science Framework [39]. Five of the R-Markdown files are for analysis of Experiment I, II, III Short, III Long, and the pooled data. The eighth file is for graphics generation. The second-to-last file takes a sample of ten random participants from the first experiment for every protocol and builds the base model from this subset. This sample analysis is to show that the main dependencies of wavelength and series can come from a smaller sample. An html file with the same file name shows an example. The last file is for calculating estimates for prereceptoral filtering and using those estimates to calculate irradiance and photon density values from the spectral irradiance measurements. (ZIP) [file pone.0253030.s017.zip › subset_of_10_for_experiment_I.html]

Pupil results - Generalized Additive Mixed Models


# Pupil results - Generalized Additive Mixed Models

This file generates generalized additive (mixed) models from the data with a sample size of 10 out of the available participants.

```
## ── Attaching packages ─────────────────────────────────────── tidyverse 1.3.0 ──
```

```
## ✓ ggplot2 3.3.5     ✓ purrr   0.3.4
## ✓ tibble  3.1.2     ✓ dplyr   1.0.7
## ✓ tidyr   1.1.3     ✓ stringr 1.4.0
## ✓ readr   2.0.0     ✓ forcats 0.5.1
```

```
## ── Conflicts ────────────────────────────────────────── tidyverse_conflicts() ──
## x dplyr::filter() masks stats::filter()
## x dplyr::lag()    masks stats::lag()
```

```
## 
## ********************************************************
```

```
## Note: As of version 1.0.0, cowplot does not change the
```

```
##   default ggplot2 theme anymore. To recover the previous
```

```
##   behavior, execute:
##   theme_set(theme_cowplot())
```

```
## ********************************************************
```

```
## 
## Attaching package: 'lubridate'
```

```
## The following object is masked from 'package:cowplot':
## 
##     stamp
```

```
## The following objects are masked from 'package:base':
## 
##     date, intersect, setdiff, union
```

```
## Loading required package: nlme
```

```
## 
## Attaching package: 'nlme'
```

```
## The following object is masked from 'package:dplyr':
## 
##     collapse
```

```
## This is mgcv 1.8-31. For overview type 'help("mgcv-package")'.
```

```
setwd(dirname(rstudioapi::getActiveDocumentContext()$path)) # set working directory to script directory

Date_of_Data <- "2021-01-08"

data <- read.csv(here::here("data_output", "general", paste("WL_average_", Date_of_Data ,".csv", sep = ""))) # load in the file with average of last 5 Seconds of every light step

#creating a subset with equal numbers of Ups and Downs

sample <- data  %>% dplyr::filter(Dir == "Up" | Dir == "Down") %>% group_by(Dir) %>% select(Code) %>% unique()
```

```
## Adding missing grouping variables: `Dir`
```

```
sample <- slice_sample(sample, n = 10)
sample
```

```
data_sub_up <- data %>% dplyr::filter(Dir == "Up") %>% filter(Code %in% sample$Code[sample$Dir == "Up"])
data_sub_down <- data %>% dplyr::filter(Dir == "Down") %>% filter(Code %in% sample$Code[sample$Dir == "Down"])

n_up <- data_sub_up %>% dplyr::filter(Dir == "Up") %>%  select(Code) %>% unique()
n_up <- length(n_up$Code)

n_down <- data_sub_down %>% dplyr::filter(Dir == "Down") %>%  select(Code) %>% unique()
n_down <- length(n_down$Code)

data <- rbind(data_sub_up, data_sub_down)

data <- add_row(data, Code = "Pred", Dir = "Timeless")
data$Code <- as.factor(data$Code) 
data$Dir <- as.factor(data$Dir)
data$Sex <- as.factor(data$Sex)
data <- data[-nrow(data),]
```

```
pup_mod <- bam(Amplitude ~ s(Index) + s(Wavelength, k = 12) + s(Code, bs="re") + s(Wavelength, by = Code, m=1) + log10(Energy), 
                data = data, method = "fREML", family = scat, 
                control= list(nthreads=2), discrete = TRUE, drop.unused.levels=FALSE)

plot(pup_mod, select = 2, shade = TRUE, shift = coef(pup_mod)[1], rug = F, seWithMean = TRUE)
```

```
plot(pup_mod, select = 1, shade = TRUE, shift = coef(pup_mod)[1], rug = F, seWithMean = TRUE)
```

```
summary(pup_mod)
```

```
## 
## Family: Scaled t(3,2.99) 
## Link function: identity 
## 
## Formula:
## Amplitude ~ s(Index) + s(Wavelength, k = 12) + s(Code, bs = "re") + 
##     s(Wavelength, by = Code, m = 1) + log10(Energy)
## 
## Parametric coefficients:
##               Estimate Std. Error t value Pr(>|t|)    
## (Intercept)     49.383      4.183  11.805  < 2e-16 ***
## log10(Energy)    8.690      2.464   3.527 0.000438 ***
## ---
## Signif. codes:  0 '***' 0.001 '**' 0.01 '*' 0.05 '.' 0.1 ' ' 1
## 
## Approximate significance of smooth terms:
##                               edf Ref.df       F  p-value    
## s(Index)                    1.911  2.023  10.415 2.20e-05 ***
## s(Wavelength)               2.430  2.700   8.384 2.51e-05 ***
## s(Code)                    16.070 18.000 238.921  < 2e-16 ***
## s(Wavelength):CodeAB030302  7.219  8.000  12.533  < 2e-16 ***
## s(Wavelength):CodeAC020403  7.181  8.000   7.778 1.87e-14 ***
## s(Wavelength):CodeAE021103  7.156  8.000  15.060  < 2e-16 ***
## s(Wavelength):CodeAI0807    6.793  8.000   3.317 5.53e-06 ***
## s(Wavelength):CodeAO1105    7.267  8.000  24.741  < 2e-16 ***
## s(Wavelength):CodeBK120501  6.972  8.000   4.678 3.84e-07 ***
## s(Wavelength):CodeBK120504  7.193  8.000   5.570 7.49e-09 ***
## s(Wavelength):CodeFH0610    7.068  8.000  38.419  < 2e-16 ***
## s(Wavelength):CodeGM070201  5.838  8.000  22.836  < 2e-16 ***
## s(Wavelength):CodeHB1012    7.013  8.000   2.462 0.000459 ***
## s(Wavelength):CodeHG0712    6.783  8.000   8.871 1.45e-10 ***
## s(Wavelength):CodeHU0210    7.054  8.000   1.962 0.015444 *  
## s(Wavelength):CodeKG110502  7.138  8.000  12.257  < 2e-16 ***
## s(Wavelength):CodeMI0703    7.068  8.000   5.346 2.47e-09 ***
## s(Wavelength):CodeMK120501  6.856  8.000  22.017  < 2e-16 ***
## s(Wavelength):CodeNA0910    6.932  8.000  11.440 5.48e-08 ***
## s(Wavelength):CodePG091001  6.728  8.000   9.578  < 2e-16 ***
## s(Wavelength):CodePred      0.000  0.000      NA 1.000000    
## s(Wavelength):CodeTR0503    6.600  8.000   7.413 1.32e-14 ***
## s(Wavelength):CodeVL0411-J  7.013  8.000   2.430 0.001265 ** 
## ---
## Signif. codes:  0 '***' 0.001 '**' 0.01 '*' 0.05 '.' 0.1 ' ' 1
## 
## Rank: 194/202
## R-sq.(adj) =  0.881   Deviance explained = 77.6%
## fREML =    Inf  Scale est. = 1         n = 1220
```
