# Supplementary material for: Spectral dependency of the human pupillary light reflex. Influences of pre-adaptation and chronotype
Source: PLoS One. 2022 Jan 12;17(1):e0253030. doi: 10.1371/journal.pone.0253030 (PMC8754338; doi:10.1371/journal.pone.0253030)
Supplement: S4 File — The zip file contains three files. One html file with a scalable and rotatable diagram of nPC (denoted as “Amplitude”) vs. wavelength and time. An html viewer, or browser with support for html widgets, is needed. The other two files are snapshots from the html file for each condition of Dark. Color mapping is according to the z-axis (nPC) for better visibility. (HTML) [file pone.0253030.s018.html]

supplemental Figure S9


supplemental Figure S9

# Experiment 2

## Row

### Without periods of darkness between light steps

### With periods of darkness between light steps
